# Specific anatomy and radiographic illustration of the digestive tract and transit time of two orally administered contrast media in Inland bearded dragons (*Pogona vitticeps*)

**DOI:** 10.1371/journal.pone.0221050

**Published:** 2019-08-22

**Authors:** Karina A. Mathes, Katharina Radelof, Elisabeth Engelke, Karl Rohn, Christiane Pfarrer, Michael Fehr

**Affiliations:** 1 Department of Reptiles and Amphibians, Clinic for Small Mammals, Reptiles and Birds, University of Veterinary Medicine, Hannover, Germany; 2 Institute for Anatomy, University of Veterinary Medicine, Hannover, Germany, University of Veterinary Medicine Hannover, Hannover, Germany; 3 Institute for Biometry, Epidemiology and Information Processing University of Veterinary Medicine, Hannover, Germany; Colorado State University College of Veterinary Medicine and Biomedical Sciences, UNITED STATES

## Abstract

The aim of this study was to describe the specific gross and radiographic anatomy of the digestive tract of inland bearded dragons (*Pogona vitticeps*). Eleven bearded dragon cadavers of both sexes (6 females, 5 males) were dissected to examine, measure, and document the specific gross anatomy of the alimentary canal. Measurements collected from the cadavers included snout-vent length, total length of the alimentary canal, and the lengths of the individual sections of the gastrointestinal tract, including the esophagus, stomach, small intestine, ampulla coli, isthmus coli, rectum, and the distance from the coprodeum to the vent opening. Twenty-two healthy adult bearded dragons (13 females, 9 males) maintained under standardized husbandry conditions underwent a physical examination, blood collection, and whole-body dorsoventral and lateral survey radiographs; these animals were used to provide the radiographic images of the complete digestive tract. For the subsequent contrast passage studies, two different contrast media, barium sulfate (BaSO_4_, Barilux suspension) and an iodinated ionic radiocontrast agent (Sodium meglumine amidotrizoate [SMAT], Gastrografin), were used. Water-diluted Barilux suspension (dose 9 ml/kg) was administered orally to 5 bearded dragons, while Gastrografin (dose 5ml/kg) was administered orally to 21 bearded dragons. Four animals were used for both contrast media studies, but received a break of four weeks in between. Dorsoventral and laterolateral radiographs were collected at 0 (baseline), 15, 30, and 45 minutes and 1, 2, 3, 4, 5, 6, 8, 10, 12, 24, 30, and 36 hours after each contrast medium was administered. Both contrast media were found to illustrate the alimentary tracts in the adult bearded dragons. Transit time was substantially faster with SMAT, and SMAT illustrated the entire gastrointestinal tract within 36 hours; BaSO_4_ did not fully illustrate the gastrointestinal tract in 36 hours. These results might serve as a guideline for the interpretation of subsequent contrast studies in this lizard species.

## Introduction

Central inland bearded dragons (*Pogona vitticeps*) are popular pets that are routinely presented to veterinarians for medical and surgical care [1, 2]. Due to the stoic temperament of these animals, early detection of diseases can be challenging. For this reason, it is important that appropriate diagnostics, such as imaging techniques, are selected to investigate the status of these animals. Unfortunately, there is a general lack of evidence-based knowledge regarding basic anatomy and physiology of this species, which can make it challenging for veterinarians to interpret diagnostic tests. This is especially true for the gastrointestinal system of bearded dragons, as there are numerous diseases that can affect the alimentary canal of captive bearded dragons [3, 4]. Because of these challenges, it is important for veterinarians to develop evidence-based criteria for evaluating and interpreting the health status of bearded dragons.

Diagnostic imaging is an often underutilized tool in herpetological medicine [5]. Therefore, developing evidence-based methods for further elucidating the value of imaging diagnostics in reptiles, such as radiography [6, 7], ultrasound [2, 8–10], computed tomography [11–15] and magnetic resonance imaging [16], could prove most useful for assessing a patient’s health status [17]. In-depth knowledge of the special anatomy [18] and morphology of a particular species is needed to interpret the results of diagnostic imaging [19]. This is one of the limitations of standard radiographic techniques, as the information gained on the gastrointestinal tract may be limited [20, 21]. To increase the informative value of survey radiographs, contrast media can be used. Contrast radiography studies can be used in the diagnosis of gastrointestinal disorders of reptiles, as well as indirectly for other viscera such as the liver and reproductive tract [7]. Single- and double- contrast agents are considered as the primary diagnostic methods for imaging the stomach and bowels of wildlife and zoo animals [22]. In addition to illustrating the gastrointestinal tract, contrast media can also be used to measure gastrointestinal transit times [23, 24]. Barium sulfate is a non-iodized contrast media that has been used in reptiles, including bearded dragons [25, 26], green iguanas (*Iguana iguana*) [27], ball pythons (*Python regius*) [28], leopard tortoises (former *Testudo pardalis*) [29], Arrau or Giant South American turtles (*Podocnemis expansa*) [30], and West African Mud turtles (*Pelusios castaneus*) [31]. Sodium meglumine amidotrizoate (SMAT, Gastrografin), a water-soluble, hyperosmolar, nonionic iodinate contrast medium, has been used in leopard geckos (*Eublepharis macularius*) [32], Greek tortoises (*Testudo hermanni*) [33], and in a comparison study with barium sulfate in red-eared slider turtles (*Trachemys scripta elegans*) [34], Greek tortoises [35] and loggerhead sea turtles (*Caretta caretta*) [36]. Cited studies suggest that these contrast media (such as barium sulfate and sodium meglumine amidotrizoate) appear to be safe in reptiles and that they could be used to develop evidence-based data for clinicans to illustrate the specialties of a speciess’ gastrointestinal tract.

The aims of this study were to obtain anatomical reference data of the alimentary canal of bearded dragon and to compare two oral contrast media, barium sulfate and SMAT, in regards to the ease of administration, transit time, and image quality. The specific hypotheses being tested were that: 1) both barium and SMAT will highlight the alimentary canal of the bearded dragon, and that 2) SMAT will be the preferred contrast because of transit time and image quality.

## Materials and methods

### Anatomic examination of cadavers

Gross anatomic examinations of the alimentary canals of 11 (6 female, 5 male) adult bearded dragons were performed for this study. The animals used for this study had died spontaneously or were humanely euthanized because of severe illness or injury; the cause of death did not affect the gastrointestinal tract. Until the examination, the cadavers were stored at -18°C. After the cadavers were thawed, the ventral wall of the body cavity was incised in a cranial to caudal direction along the median to expose the viscera. The topographies of the various sections of the alimentary canal were observed in situ and photographs collected (Panasonic DMC-FZ150, Panasonic Corporation, Kadoma Osaka Japan). Once the photographs were collected, the alimentary tract was removed completely. Each section of the tract was identified and measured. The lengths of the various sections of the alimentary tract as well as the total length of the digestive tract were set in relation to the snout-vent length (SVL) and displayed as ratio. The alimentary canal was opened lengthwise, and any contents removed by rinsing with tap water. The macroscopic features were noted and photographed.

Prior to removing and opening the alimentary tract, the cadavers were radiographed with the viscera in situ to use as a baseline for characterizing the radiographic positioning of the alimentary tract. Survey radiographic images were obtained from each dragon in the dorsoventral and lateral positions (Gierth HF 400 High frequency diagnostic x-ray system, GIERTH X-Ray International GmbH, Riesa, Germany). For the dorsoventral projection, all lizard cadavers were positioned in ventral recumbency. Normal anatomic body position (ventral recumbency) was maintained for the laterolateral projection using a horizontal beam by rotating the x-ray tube 90° and positioning the animal cadavers on a standardized foam pad. Radiographic exposure settings were standardized for each animal, including a focal film distance (FFD) of 60 cm, voltage of 42 kV, and length of time of the electrical current varying between 1.96 and 2.05 mAs. Imaging data were stored on an optical disk, and data were analyzed and digitized with a dataprocessing unit (Processor CR35-X; AGFA HealthCare GmbH, Bonn, Germany) using mammography imaging plates (Mammography Film CRMM 3.0 Extremities; AGFA HealthCare GmbH, Bonn, Germany).

### Examination of live animals- contrast studies

Twenty-two adult bearded dragons (13 females, 9 males) were examined for the second aim of the study. Animals recruited for the study had to be >100 g in body weight and clinically healthy. Five animals were used for a barium sulfate (BaSO_4_, Barilux suspension, manufacturer information?) contrast study and 21 animals for a SMAT contrast study. Four of the 22 animals were used in both contrast media studies; however, a four week washout was provided between studies. The lizards were owned by individual households or zoological parks and were part of various collections in Lower Saxony, Germany. Written consent was obtained from each client and zoological park prior to enrolling the bearded dragon into this study.

The study was carried out in strict accordance with the recommendations from the Institutional Animal Care and Use Committee at the University of Veterinary Medicine Hanover, Germany, and was also approved by the Lower Saxony State Office for Consumer Protection and Food Safety, the regional office responsible for oversight of animal research (Protocol Number 33.9-42502-05-14A457).

Bearded dragons were housed alone or in pairs in their respective home or zoological park. The diets offered were comprised of lettuce and mixtures of various vegetables, offered daily, and live crickets or locusts, offered once to twice a week. At least 24 to 48 hours before the lizards were examined, they were transported to the Clinic for Small Mammals, Reptiles, and Birds (University of Veterinary Medicine Hannover) and housed in individual glass terrariums maintained in a separate room with an ambient temperature of 24° to 26°C. Temperatures within the terrarium were controlled by means of spot heat lamps (hot spot temperature up to 40°C) and light was provided by daylight and fluorescent tubes (lighting duration 7 am– 7 pm). Each terrarium contained a cardboard box for a shelter and was coated with pulp to provide the lizard an opportunity to climb. Animals were fed a mixture of various vegetables and salads once daily in the morning, and no insects were provided during their stay in the clinic. The bearded dragons were bathed in tap water each morning and provided ultraviolet-light (Osram Ultra Vita Lux, 300 W, OSRAM GmbH, Augsburg, Germany) for 20 minutes as part of their in-clinic routine.

A physical examination was performed on each bearded dragon to determine it was healthy. The physical examinations included determination of the sex and inspection and/or palpation of the skin and scales, oral cavity, eyes, nares, tympanic membranes, skeleton, extremities, muscle tone, coelomic cavity, cloaca, and tail. Body weight was measured to the nearest gram. Snout-vent length (SVL) was measured to the nearest mm using a measuring tape from the rostral tip of the snout to the aboral end of the cloaca.

A blood sample of 0.7–1 mL was collected from the ventral tail vein (vena coccygea ventralis) using a 22 gauge needle fastened to a 1 ml syringe. The blood samples were immediately transferred into lithium heparin coated tubes for biochemical analyses and microhematocrit tubes to measure the packed cell volume (PCV). Following measurements of the PCV, 19 biochemical analytes were measured, including alanine aminotransferase (ALT), glutamate dehydrogenase (GDH), alkaline phosphatase (ALP), aspartate aminotransferase (AST), cholinesterase, creatine kinase (CK), blood urea nitrogen (BUN), sodium, potassium, total calcium, ionized calcium, phosphorus, fructosamine, cholesterol, glucose, uric acid, albumin, total protein (TP), and total bilirubin. The concentrations of the electrolytes (sodium, potassium, ionized calcium) were measured using a fully automated blood gas analyzer system (Rapidlab 1200 system, Siemens Healthcare Diagnostics GmbH, Eschborn, Germany), while the activities of the enzymes and concentrations of the other biochemistries were measured with a clinical chemistry analyzer (Cobas C 311 analyzer, Roche Diagnostics Deutschland GmbH, Mannheim, Germany).

All of the lizards were housed under a standardized daily temperature regime and examined at the same time every day. This was done to minimize any bias that might impact gastrointestinal transit times. Environmental, core, and surface body temperatures were measured 12 times per animal, including at baseline prior to the first radiograph and at 2, 3, 4, 5, 6, 8, 10, 12, 24, 30, and 36 hours after administration of the contrast media. Ambient temperatures within the terrarium and surface body temperatures were measured using a Voltcraft Infrared IR 260-8S thermometer (Conrad Electronic AG, Wollerau, Germany), while body core temperatures were measured via the cloaca using a Voltcraft PL-120-T2 thermometer (Conrad Electronic AG, Wollerau, Germany).

Survey radiographic images were obtained from each dragon in both dorsoventral and lateral projections using the same digital system used in the cadaver study. For the dorsoventral projection, all lizards were positioned in ventral recumbency directly on the imaging- plates without any physical restraint. Normal anatomic body position (ventral recumbency) was maintained for the laterolateral projection using a horizontal beam by rotating the x-ray tube 90° and positioning the unrestrained animals on a standardized foam pad. Image processing and storage were similar to the techniques described for the cadaver study.

Subsequent, 22 adult animals were selected to complete the follow-up contrast studies. Similar to the baseline study, the contrast agent studies were conducted under the same defined and standardized conditions. Contrast study start times were always initiated at 7 am and concluded at 7 pm the following day.

Barilux suspension (Barium sulfate, 830 mg/ml, Sanochemia Diagnostics Deutschland GmbH, Neuss, Germany) gastrointestinal transit times (GTT) were determined in five adult bearded dragons (3 females, 2 males) and Gastrografin (660 mg/ml meglumine amidotrizoate and 100 mg/ml sodium amidotrizoate; Bayer Vital GmbH Leverkusen, Germany) GTT were measured in 21 adult bearded dragons (12 females, 9 males). Barilux suspension (w/v) was diluted with water 1:1 so that the diluted suspension contained 415 mg/ml barium sulfate. Diluted barium sulfate suspension was administered per os via syringe at a dose of 9 ml/kg (0.9 ml/100g) body weight. Gastrografin was dosed per os at 5 ml/kg (0.5 ml/100g) body weight. Dorsoventral and laterolateral radiographs were taken at 0 min, 15 min, 30 min, 45 min, 1 hr, 2 hrs, 3 hrs, 4 hrs, 5 hrs, 6 hrs, 8 hrs, 10 hrs, 12 hrs, 24 hrs, 30 hrs and 36 hrs postcontrast administration.

A categorical scale was created to describe the six different levels of gastrointestinal filling in the living animals over time. The coded scale included the following parameters: „0”, empty pre-contrast (before filling with any contrast medium); „1”, initial filling (partially filled with contrast medium); „2“, complete filling (completely filled with contrast medium); „3“, depleting filling (decreasingly filled with contrast medium); „4“, remaining quantity (containing a residual amount of contrast media); and „5“, empty post-contrast (after filling with contrast medium—post-filling).

For each animal, new variables were calculated from these categorical scaled data for assessment of different passage duration of contrast media per organ of the GI tract: TP1 is the first time point of measuring of completely filled (first level 2), TP2 is the last time point of measuring completely filled (last level 2). Difference of TP1 and TP2 (TP2-TP1) is the duration (retention period) of complete filling (level 2) of contrast media for each single part of the GI tract.

TP3 is the first time point of beginning of filling (first level 1), TP4 is the last time point of remaining quantity (level 4) of contrast media. Difference of TP3 and TP4 (TP4-TP3) is the duration (retention period) of any contrast media (level 1 to level 4) for each single part of the GI tract.

### Statistical analysis

Two sets of data were used for the statistical analysis; data of 11 dissected animals and data of 22 living animals for the contrast studies with barium sulfate and SMAT. The distributions of the data were evaluated using the Shapiro-Wilk test and visual assessment of model residuals. Normally distributed data are reported by the mean, standard deviation (SD), and minimum-maximum (min-max) values, while non-normal data are reported by the median, 10–90 percentiles (%), and min-max. Log-normal data (right skewed) were log transformed for parametric testing. An independent samples t-test was used to compare differences in morphometrics by sex for normally distributed data. Levene’s test was used to evaluate the data for equality of variance.

A two-way analysis of variance (ANOVA) with repeated measurements was calculated for analyzing the effect of the two contrast media in the course of 36 hours (with 12 measuring time points) for body surface temperatures and cloacal temperatures.

Due to not normal distribution, differences between contrast-media of TP1, TP3 and retention periods (TP2-TP1 and TP4-TP3) were tested with Wilcoxon-2-Sample-test. A p<0.05 was used to determine statistical significance. During statistical procedures, unequal sample sizes of the two contrast media were taken into account. Statistical analyses were performed with the use of statistical analysis system software (SAS, Version 9.4, SAS Institute, Cary, USA).

## Results

### Macroscopic anatomy of bearded dragons

#### Topography of the different parts of the alimentary canal

The coelom of bearded dragons starts cranially with the thoracic inlet (*apertura thoracis cranialis*) and ends caudally with the pelvic outlet (*apertura pelvis caudalis*). The dorsal skeletal frame of the coelom is the vertebral column, which includes 13 thoracic vertebrae, five lumbar vertebrae, and three sacral vertebrae that are fused and form the sacral bone. The ribs extend ventrolaterally from the thoracic vertebral column and form the widest part of the trunk at their ventral ends. The ventral ends of the last pair of ribs are located directly cranial to the pelvis. From the cranioventrally located sternum, 4–5 pairs of sternal ribs extend on both sides dorsocaudally until the level of the 5 th and 6th vertebral rib pair.

**Fig 1:**
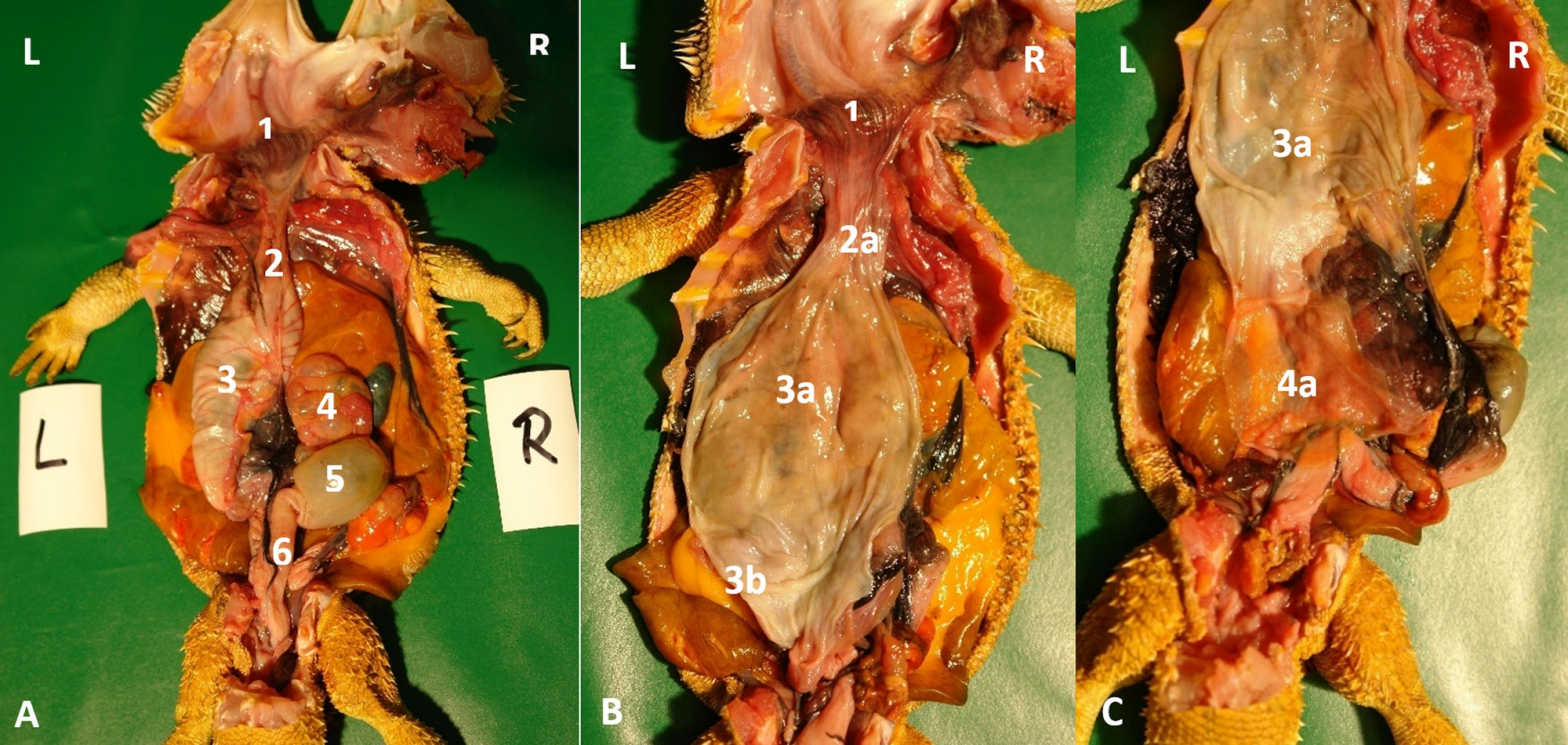
Topography of the alimentary canal of a female bearded dragon, dorsal view. Dorsal skin, muscles, skeleton, and serosa removed; lungs distracted cranially; and dorsal pelvis removed for an in situ view. L: left; R: right. **A.** Topography in situ: oral cavity and pharynx (1), esophagus (2), stomach (3), small intestine (4), ampulla coli (5), and rectum (6). **B.** Alimentary canal opened: oral cavity and pharynx (1), esophagus (2a), stomach (3a), and transverse gastric fold caused by angular incision (3b). **C.** Alimentary canal opened and distracted cranially to demonstrate the pyloric circular fold (3c*); stomach (3a), and duodenum (4a).

**Fig 2:**
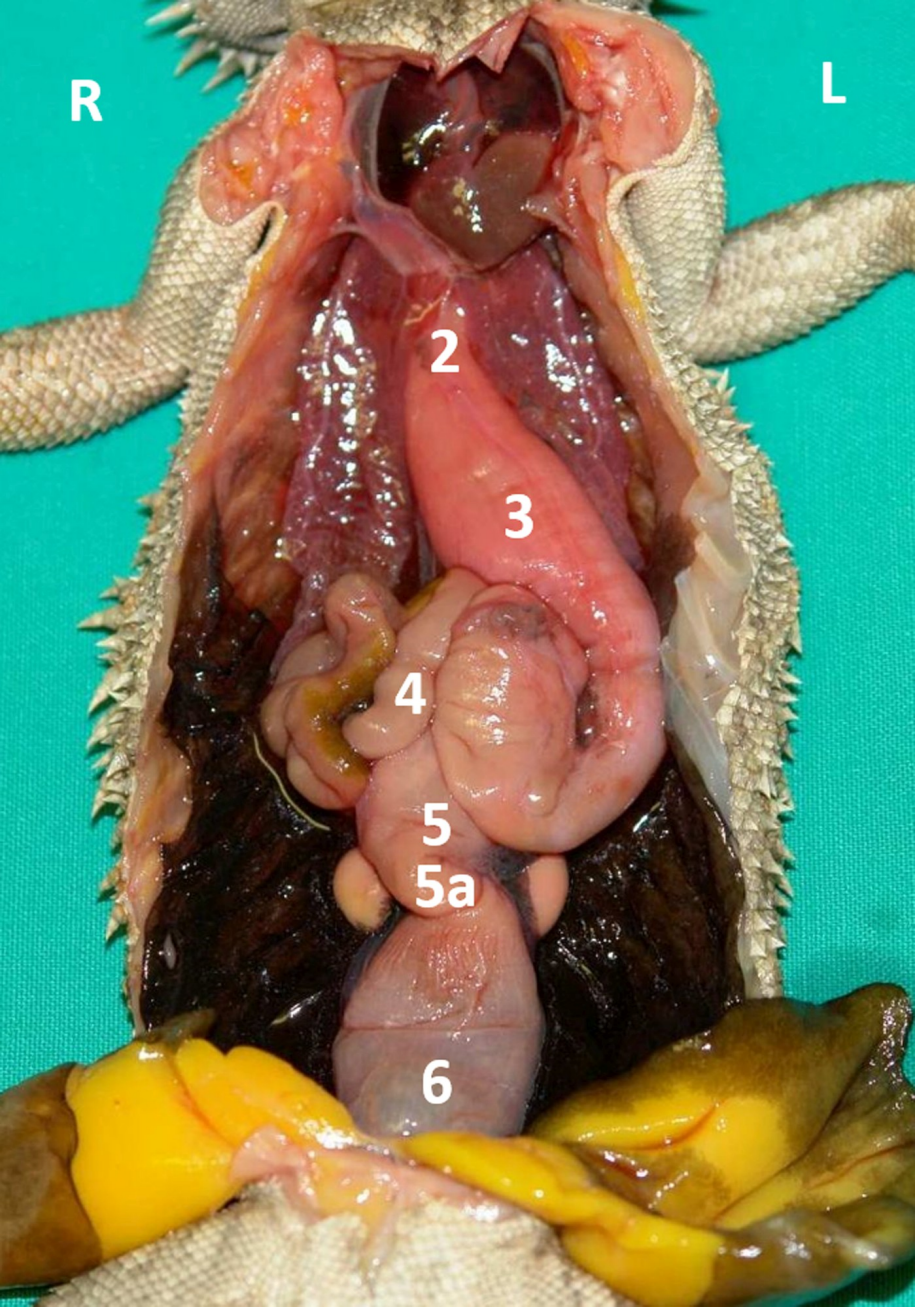
Topography of the alimentary canal of a male bearded dragon in situ, ventral view. Ventral skin, muscles, skeleton, and serosa removed; pericardium opened ventrally and partially removed; and fat bodies distracted caudolaterally to expose the esophagus (2), stomach (3), small intestine (4), ampulla coli (5), isthmus coli (5), and rectum (6).

Caudal to the combined oral and pharyngeal cavities (Fig 1A/1 and 1B/1), the esophagus (Fig 1A/2 and 1B/2a) is the first organ of the digestive tract; it is located completely inside the coelom. The esophageal mucosa has macroscopically visible longitudinal folds (Fig 1B/2a). In the caudal direction, the esophagus travels along midline and merges into the stomach (Fig 1A/3; 1B/3a; 1C/3a and 1/3) along the left side of the coelom, with the transition located between the 7^th^ and 9^th^ rib pair. There is no macroscopically distinct border between these two organs (compare Fig 1B/2a and 3a). In bearded dragons, the stomach, or ventriculus, (Fig 1A/3, 1B/3a and 2/3) is shaped like an elongated C, of which the longer section, the body of the stomach, is located on the left side of the coelom and the shorter pyloric section more to the right side. The small curvature is oriented cranially and the large curvature extends caudally in the coelom. The lumen of the pyloric section of the stomach narrows distinctly and is directed cranially from left- midline to right-midline. It terminates with the clearly visible pylorus, and its wall contains the sphincter pylori muscle. Macroscopically, the stomach´s inner relief is primarily smooth-walled with some longitudinal folds along its length (Fig 1B/3a and 1C/3a). When opening the stomach, a transverse fold is visible at the small curvature at the beginning of the distal third of the stomach. This is the inner relief of the angular incision as the border to the pyloric section (Fig 1B/3b). At the pylorus, a circular fold is clearly visible (Fig 1C/3c*). Subsequently, the stomach merges into the duodenum (Fig 1C/4a). Because of its comparatively long mesentery (up to 6 cm), the position of the small intestine is relatively flexible and it can be found on either the left or right side of the coelom. Most of the small intestine is located in the caudal half of the coelom; however, if there are a large number of follicles or eggs in the salpinx or pronounced fat bodies, the small intestine may be displaced into the cranial- half of the coelom. The lining of the small intestine is comprised of longitudinal folds, which vary in height. There are three defined sections of the small intestine (Fig 1A/4 and 2/4), the duodenum, jejunum, and ileum. The duodenum disembogues the common bile duct and the pancreatic duct. Furthermore, the distal section of the pancreas lies adjacent to the proximal section of the duodenum in its mesentery. The jejunum is the longest section of the small intestine. The ileum, which has a smaller lumen and is lighter in color than the jejunum, terminates with a clearly visible border. The large intestine is comprised of the colon and the rectum. The colon has two sections, a colic ampulla and a colic isthmus. The first section, the colic ampulla (Fig 1A/5 and 2/5), is spherical in shape. In some of the dissected animals (27%, 3/11), a tiny blind diverticulum could be observed protruding dorsally from the ampulla coli and appeared to be a rudimentary cecum. The colic ampulla is located on the right, caudal section of the coelom and is attached to the duodenum with a short peritoneal plica. It narrowed toward the short and thick-walled second section of the large intestine, the colic isthmus (Fig 2/5). Its wall contains a strong muscular layer. The rectum (Fig 1A/6 and 2/6) showed a distinct enlargement of the lumen compared to the colic isthmus and is directly ventral and parallel to the vertebral column. The mucosa of the colic ampulla possesses high longitudinal folds, which decrease at the colic isthmus and increase significantly again in the rectum. The border between the rectum and the coprodeum, the first section of the cloaca, is marked by a distinct circular fold of the mucosa. The cloaca has further distinct circular folds subdividing it into the coprodeum, urodeum, and proctodeum. The longitudinal folds in the cloaca are small. The cloaca terminates at the vent, which contains the sphincter cloacae (muscle). Examples of the complete gastrointestinal tract in situ can be found in Figs 1A–1C and 2.

#### Dimension and measurements of the digestive tract of the dissected animals

Lengths and widths of the different sections and entire digestive tracts of the 11 bearded dragon cadavers were measured, as well as the snout-vent length (SVL), and the ratios of the digestive tract to the snout-vent length (RSVL) were calculated. For the dissected animals, Shapiro-Wilk test and visual assessment of quantile-plots (QQ-plots) showed no normal distribution in 73% of the data.

Therefore, median (as location parameter), 10–90 percentiles (%), min and max (as dispersion parameter) were provided for description of this data (Table 1).

**Table 1:** Data of adult bearded dragon cadavers (1–11).

		Median	10%	90%	Min	Max
Esophagus	W	0.9	0.6	1.2	0.5	1.5
	L	4.5	3.0	5.0	3.0	5.0
Stomach	W	1.9	1.5	2.4	0.7	2.8
	L	6.0	5.0	7.0	4.5	9.0
Small intestine	W	0.7	0.3	1.2	0.3	1.2
	L	21.0	16.2	32.0	14.0	36.0
Ampulla coli	W	3.0	1.8	3.5	1.4	3.5
	L	2.5	2.0	4.0	1.8	4.0
Isthmus coli	W	0.6	0.4	0.8	0.4	1.0
	L	1.0	0.7	2.0	0.6	2.0
Rectum	W	1.4	1.1	2.2	1.0	2.3
	L	6.0	4.5	8.0	3.5	8.5
Coprodeum to vent (in cm)	W	1.0	0.8	1.2	0.6	1.4
	L	1.8	1.3	2.0	1.0	2.5
Alimentary Canal (total length)		42.3	36.2	55.5	32.8	62.0
SVL (in cm)		20.5	16.8	23.0	11.8	23.5
Ratio digestive tract:SVL		2.5	1.9	3.1	1.6	3.3

Length and width of the different parts of the alimentary canal, alimentary canal length in total, snout-vent length (SVL), (all length and width specifications in cm); ratio of the digestive tract to the snout-vent length (SVL).

L = length, W = width, SVL = snout-vent length.

### Examination of live animals—Contrast studies

Body weights and SVL of the study subjects ranged between 101 and 547 g (arithmetic mean of 269 g) and SVL ranged between 15.1 and 22.7 cm (arithmetic mean 18.8 cm), respectively (Table 2).

**Table 2:** Live adult bearded dragon used in the contrast media studies.

Number	Gender	Bodyweight (in g)	Snout-vent length (in cm)
**1**	female	256	19.2
**2**	female	272	20.5
**3**	male	250	19
**4**	female	264	19.3
**5**	female	327	22.7
**6**	male	356	22.5
**7**	female	309	18.6
**8**	male	373	20.6
**9**	female	286	19.9
**10**	male	547	22.6
**11**	female	216	17.9
**12**	male	172	18.5
**13**	male	146	16
**14**	female	109	16.1
**15**	female	101	15.1
**16**	male	127	17.5
**17**	female	220	16.
**18**	male	359	18.7
**19**	female	177	17.2
**20**	male	366	19.4
**21**	female	364	19.1
**22**	female	314	17.3

Animal number, gender, bodyweight (g) and snout-vent length (SVL) (cm).

An independent t-test showed that there were no significant differences in body weight (t = 1.12, p = 0.272) or SVL (t = 1.10, p = 0.283) between male and female bearded dragons, thus the results were combined (Table 3).

**Table 3:** Descriptive statistics for the body weights and SVL for 22 bearded dragons used in the contrast study.

Parameter	Mean	SD	Min	Max
**Body weight (g)**	268.7	107.4	101.0	547.0
**SVL (cm)**	18.8	2.1	15.1	22.7

Bodyweight (g) and snout-vent length (SVL) (cm). Mean = arithmetic mean; SD = standard deviation.

Data were found to be normally distributed according to Shapiro-Wilk test and visual assessment of quantile-plots (QQ-plots). Arithmetic mean (mean), standard deviation (SD), min and max were provided for these descriptive statistics (Table 3).

### Temperature measurements

Under standardized daily temperature and light regime, temperature measurements were taken 12 times in each examined bearded dragon. These measurements consisted of temperatures of the body surface of all lizards and body core temperatures as cloacal temperatures and once before first radiographs were taken and 2, 3, 4, 5, 6, 8, 10, 12, 24, 30 and 36 hours after the administration of the contrast media to deliver a standardized procedure.

**Fig 3:**
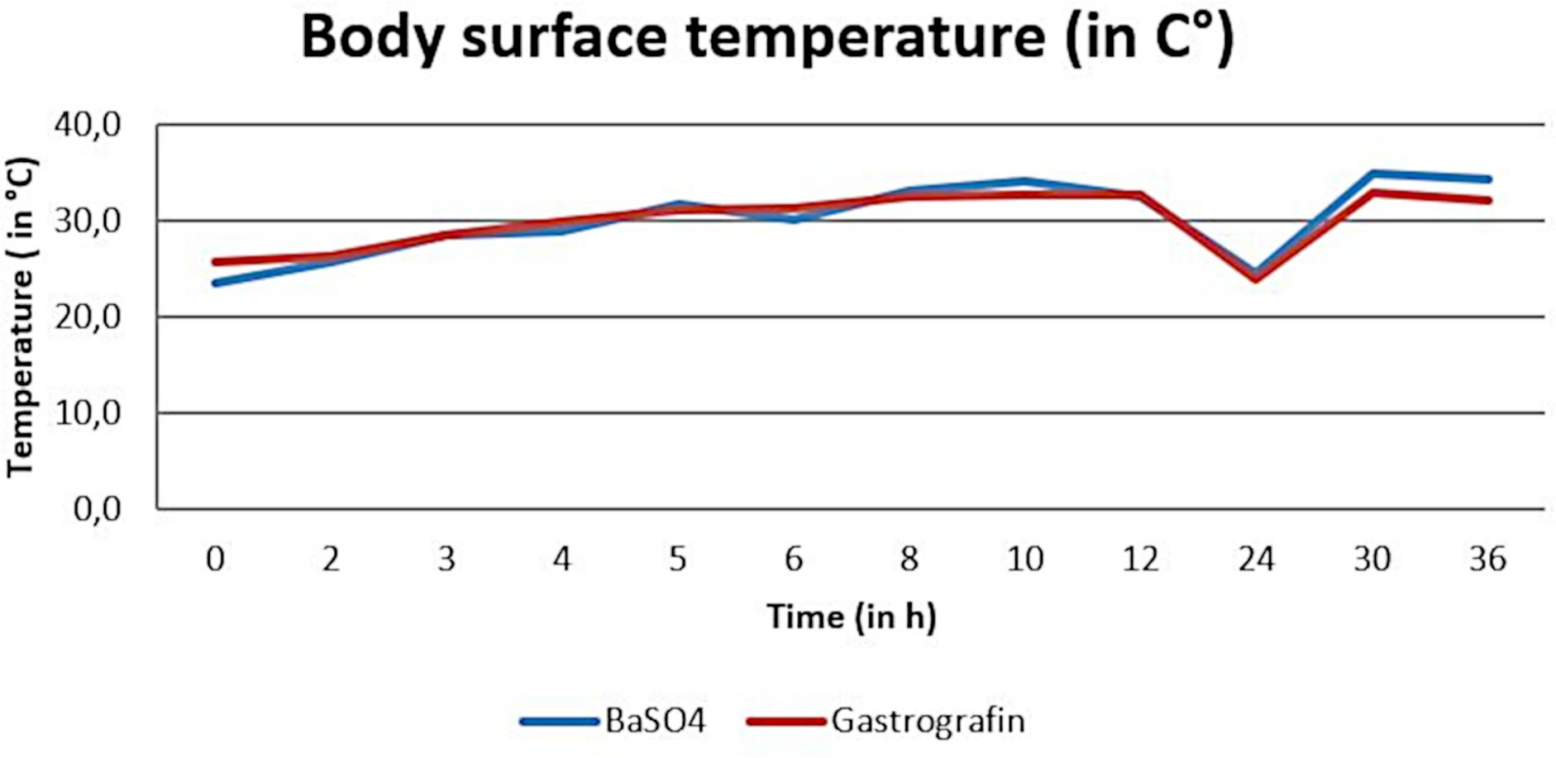
Arithmetic mean of body surface temperature (in°C) during the barium sulfate and Sodium/Meglumine–Amidotrizoate (SMAT, Gastrografin) contrast passage.

Temperature measurements during the barium sulfate and the SMAT (Gastrografin) contrast studies revealed body surface temperatures ranged between 21.5°C and 38.2°C (arithmetic mean of 30.2°C) and between 20.1°C and 39.1°C (arithmetic mean 29.9°C) (Fig 3).

**Fig 4:**
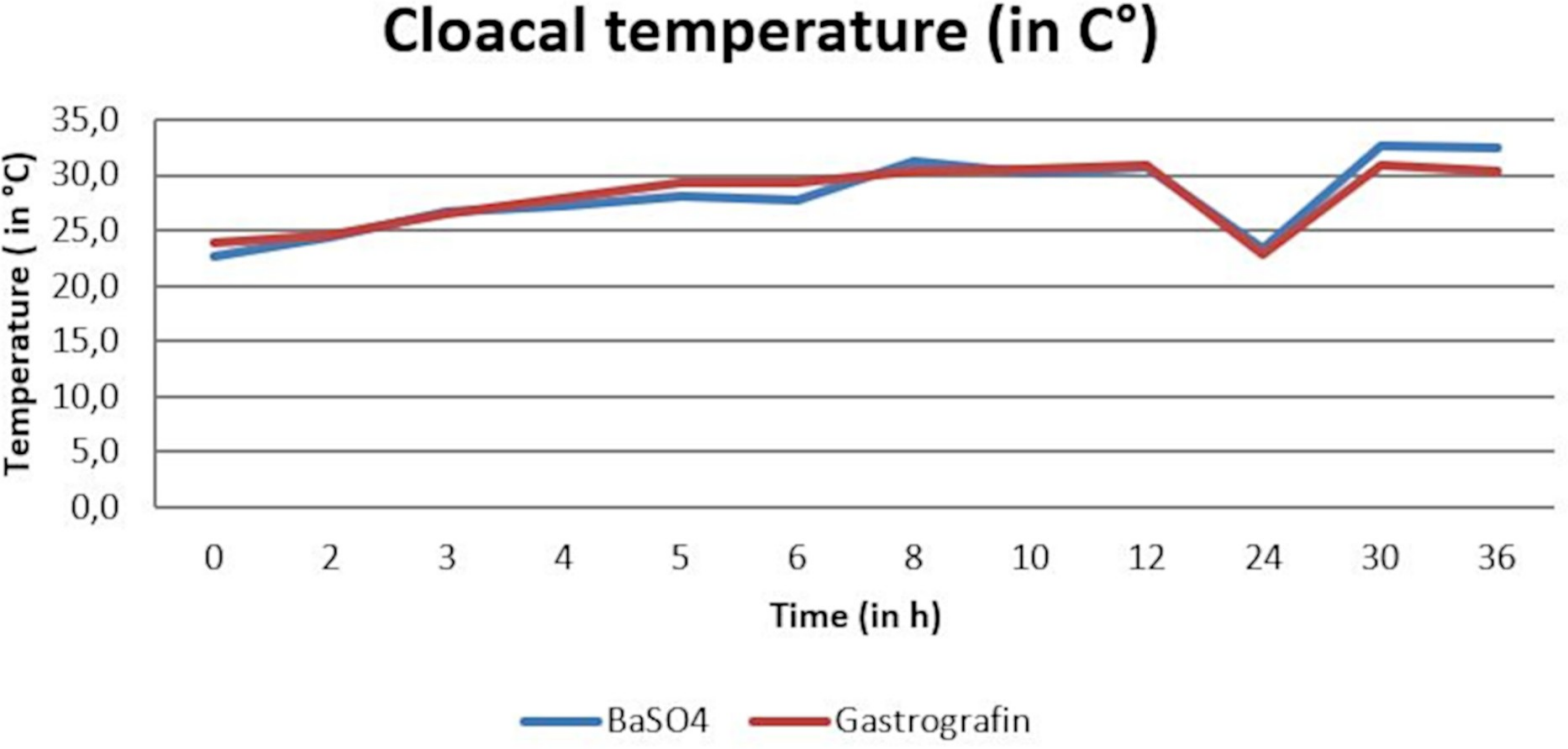
Arithmetic mean of cloacal temperature (in°C) during the barium sulfate and Sodium/Meglumine–Amidotrizoate (SMAT, Gastrografin) contrast passage.

Temperature measurements during the barium sulfate and the SMAT (Gastrografin) contrast studies revealed cloacal temperatures ranged between 22.2°C and 33.5°C (arithmetic mean of 28.1°C) and 19.8°C and 37.1°C (arithmetic mean 28.1°C). (Fig 4).

Two-way analysis of variance found no significant differences between the two contrast media during the time course (OR time period) for body surface temperature (p-value = 0.83) or cloacal temperature (p-value = 0.98). (Figs 3 and 4). There was no significant interaction between the contrast media and time.

### Radiographic contrast studies

The structures visible on the radiographs were evaluated using the anatomic knowledge gained from the cadavers. The sections of the digestive tract visible on the radiographs from the oral cavity to the cloaca included the oropharynx, esophagus, stomach, duodenum, jejunum, ileum, ampulla coli, rectum, and cloaca.

In general, the laterolateral view was essential and indispensable for the illustration of the esophagus, but the dorsoventral view is more significant for the illustration of the more caudal sections of the gastrointestinal tract.

### Barium sulfate (Barilux suspension) contrast media studies

Barium sulfate (Barilux suspension) was administered orally to five bearded dragons (3 females, 2 males). Bodyweight ranged in between 256–547 grams and snout-vent length (SVT) ranged in between 17.3–22.6 cm.

All bearded dragons initially swallowed the BaSO_4_ voluntarily; however, all five animals refused to swallow the entire dose. Two (20%) of five animals regurgitated approximately 0.05–0.15 ml of the BaSO_4_. In four (80%) of the bearded dragons, BaSO_4_ reached the ampulla coli within 36 hours; in three (60%) animals, contrast was also in the rectum by 36 hours. In one (20%) lizard, the contrast media had not passed the duodenum after 36 hours. None of the animals had contrast media in the cloaca after 36 hours. Thus, it wasn´t possible to illustrate the entire digestive tract within 36 hours of BaSO_4_ administration (Table 4 and Table 5).

**Table 4:** Time (in hours) at which barium sulfate completely filled (L2, top row) and had completely left (L5, bottom row) each segment of the gastrointestinal tract (for each single tested animal).

Contrast medium: Barium sulfate	Bearded dragon					
	1	2	3	4	5	Median (1–5)
**Oropharynx** (L 2)	0	0	0	0	0	0
**Oropharynx** (L 5)	0.5	0.75	2	0.25	1	0.75
**Esophagus** (L 2)	0	0	0.25	0	0.25	0
**Esophagus** (L 5)	0.5	1	2	0.75	1	1
**Stomach** (L 2)	0	0.75	1	0.25	2	0.75
**Stomach** (L 5)	10	4	30	>36	36	30
**Small intestine** (L 2)	2	3	24	12	10	10
**Small intestine** (L 5)	24	12	>36	>36	>36	>36
**Ampulla coli** (L 2)	10	10	30	>36	30	30
**Ampulla coli** (L 5)	>36	>36	>36	>36	>36	>36
**Rectum** (L 2)	12	24	>36	>36	36	36
**Rectum** (L 5)	>36	>36	>36	>36	>36	>36
**Cloaca** (L 2)	>36	>36	>36	>36	>36	>36
**Cloaca** (L 5)	>36	>36	>36	>36	>36	>36

Each bearded dragon 1–5 listed separately (OR individually) as well as the median of each time point and topic.

L2 = Level 2; L5 = Level 5.

**Table 5:** Course of contrast study (in hours), displaying type of contrast medium (barium sulfate). Summary of BaSO_4_ group of Table 4.

Contrast medium:	Location	Min	Max	Median
**Barium sulfate**	Oropharynx (L 2)	0	0	0
	Oropharynx (L 5)	0.25	2	0.75
**Barium sulfate**	Esophagus (L 2)	0	0.25	0
	Esophagus (L 5)	0.5	2	1
**Barium sulfate**	Stomach (L 2)	0	2	0.75
	Stomach (L 5)	4	>36	30
**Barium sulfate**	Small intestine (L 2)	2	24	10
	Small intestine (L 5)	12	>36	>36
**Barium sulfate**	Ampulla coli (L 2)	10	>36	30
	Ampulla coli (L 5)	>36	>36	>36
**Barium sulfate**	Rectum (L 2)	12	>36	>36
	Rectum (L 5)	>36	>36	>36
**Barium sulfate**	Cloaca (L 2)	>36	>36	>36
	Cloaca (L 5)	>36	>36	>36

Location within the digestive tract, minimum (Min), maximum (Max) and median (Median) time (in hours) in which each part of digestive tract was completely filled (L2, top row) and completely empty again (L5, bottom row) in bearded dragon 1–5

Min = minimum time, Max = maximum time; Median = median time; L2 = Level 2; L5 = Level 5.

#### Oropharynx and Esophagus

**Fig 5:**
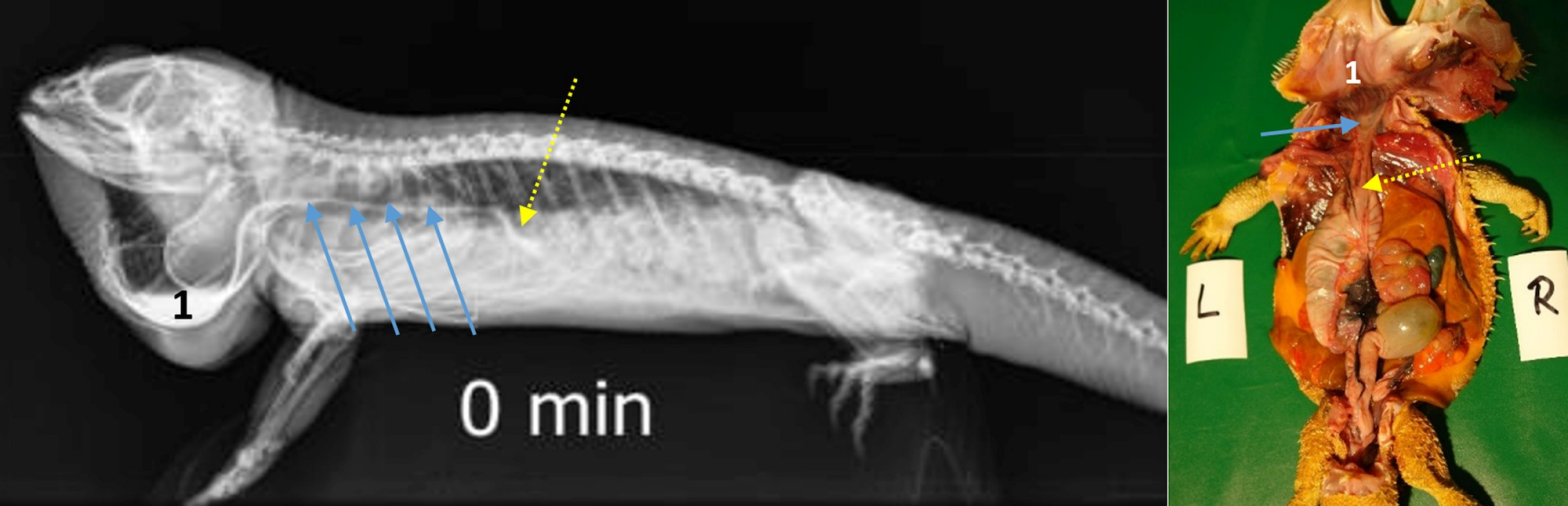
Laterolateral radiograph in ventral recumbency using a horizontal beam. Sternal recumbency on a standardized foam pad. Immediately after oral administration of BaSO_4_, (kV 42, mAs 2). Note the BaSO_4_ in the esophagus in the ventrally dependent gular area. A smaller volume of contrast media has already entered the distal esophagus (blue arrows) and is seen at the entrance (cardia) of the stomach (yellow arrow).

**Fig 6:**
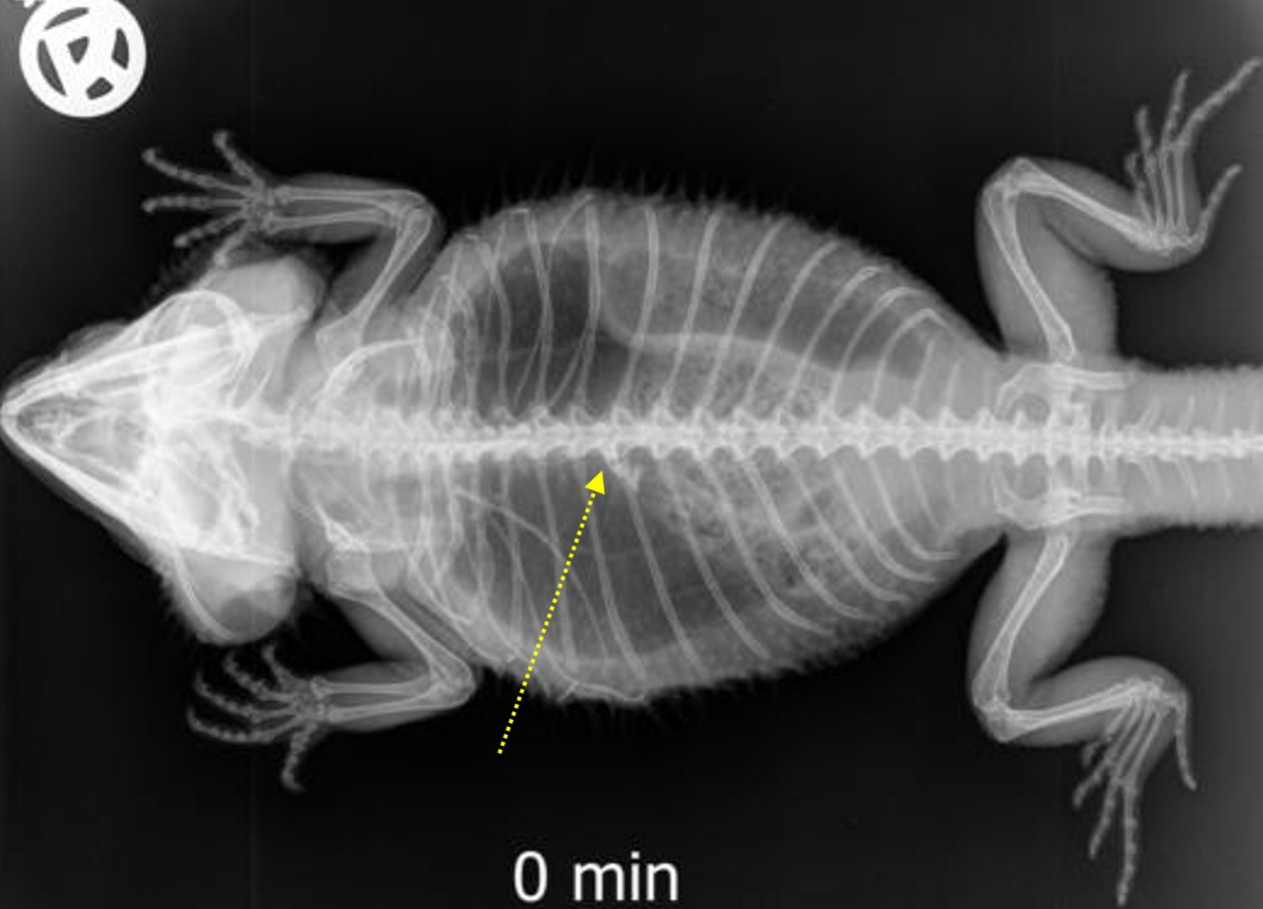
Dorsoventral (DV) radiograph immediately after oral administration of BaSO_4_. Sternal recumbency on a standardized foam pad. Immediately after oral administration of BaSO_4,_ (kV 42, mAs 2). The entrance of the stomach at the 6^th^ and 7 ^th^ thoracic vertebrae is illustrated (yellow arrow).

**Fig 7:**
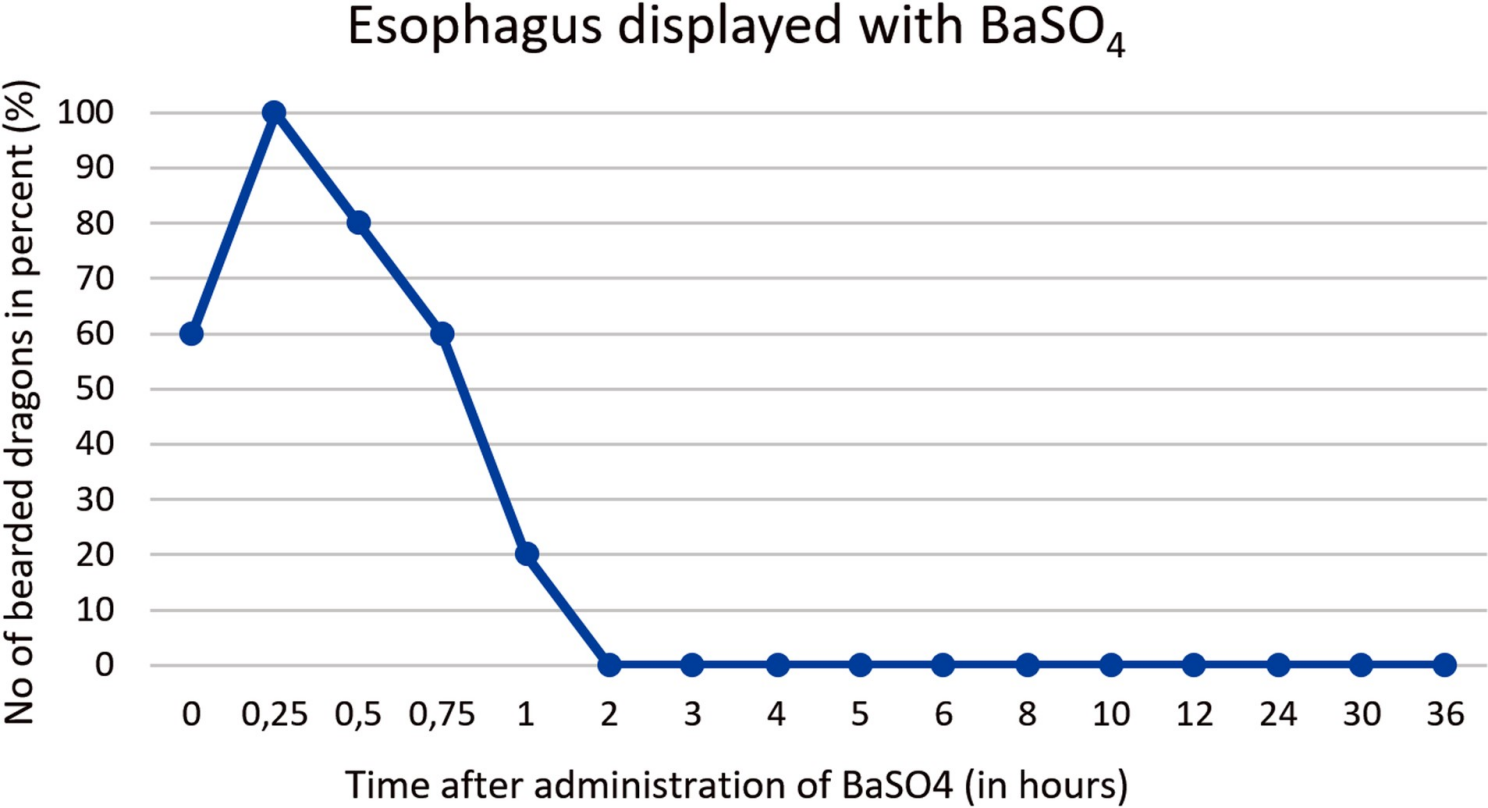
BaSO_4_ contrast visibility in the esophagus over time. Percentage of animals and frequency at measuring time point; y = number of animals in percent (%) in which contrast media could be found in the esophagus; x = time point during the barium sulfate (BaSo_4_) contrast passage (hours).

Directly after BaSO_4_ administration, and especially in the lateral view, contrast enhanced display of the oropharynx was possible in all five animals. Further, the contrast medium illustrated the esophagus within 15 minutes (Tables 4 and 5, Figs 5 and 6). Fig 7 shows the BaSO_4_ contrast passage time of the esophagus in all five bearded dragons.

BaSo_4_ completely filled the esophagus within 15 minutes and completely left the esophagus and reached the stomach within two hours in all five animals (Tables 4 and 5). The anatomic location of the esophagus could be visualized in all five animals. Longitudinal or horizontal folds were not visible in the esophagus with BaSO_4_.

#### Stomach

**Fig 8:**
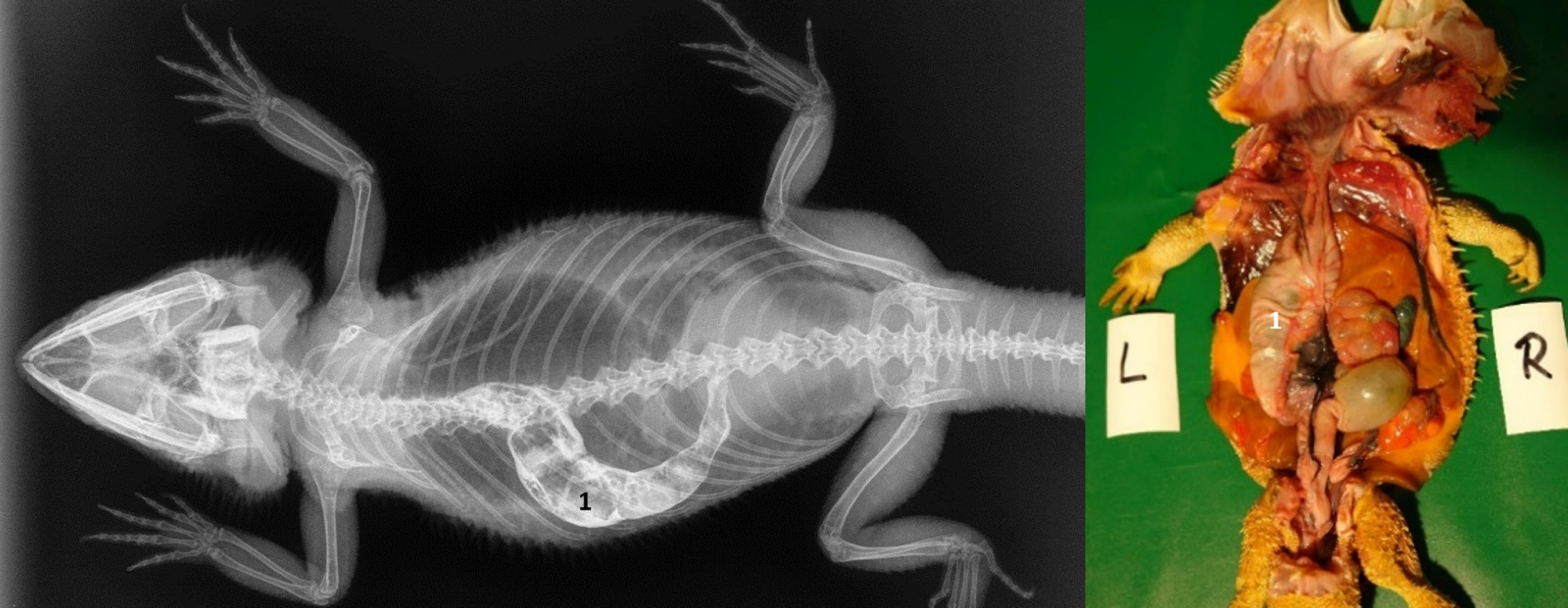
Dorsoventral (DV) radiograph 15 minutes after oral administration of BaSO_4_. 15 minutes after oral administration of BaSO_4_, (kV 42, mAs 2). The stomach (1) is visible as an elongated “C” and is primarily located on the left side of the coelom.

**Fig 9:**
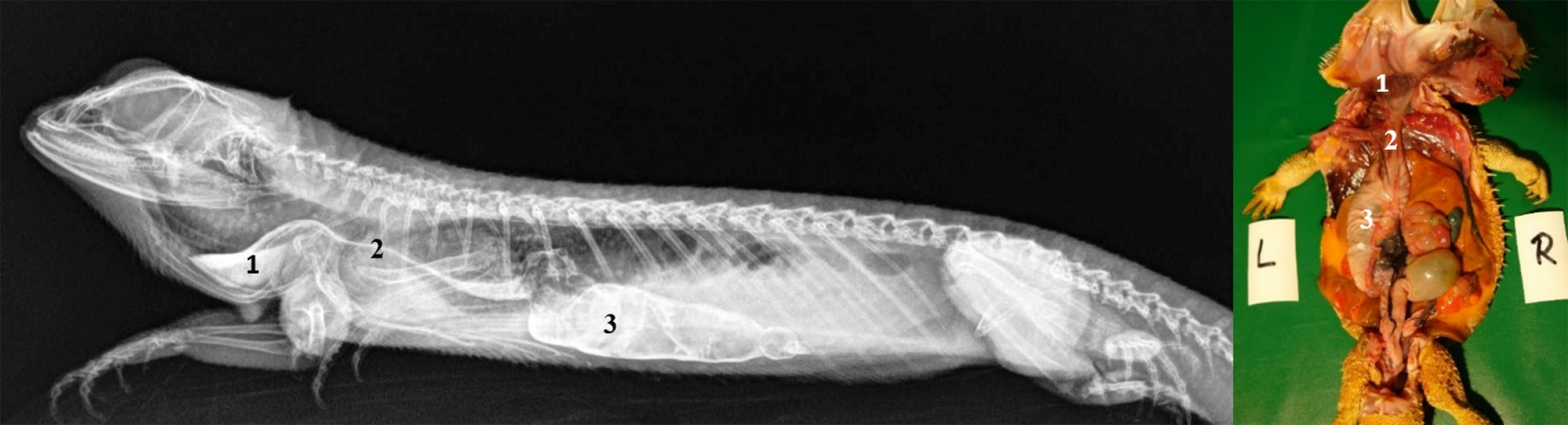
Laterolateral (LL) radiograph in ventral recumbency and using a horizontal beam. 15 minutes after oral administration of BaSO_4_, (kV 42, mAs 2). Note the BaSO_4_ in the esophagus in the ventrally dependent gular area (1), distal esophagus (2), and stomach (3).

**Fig 10:**
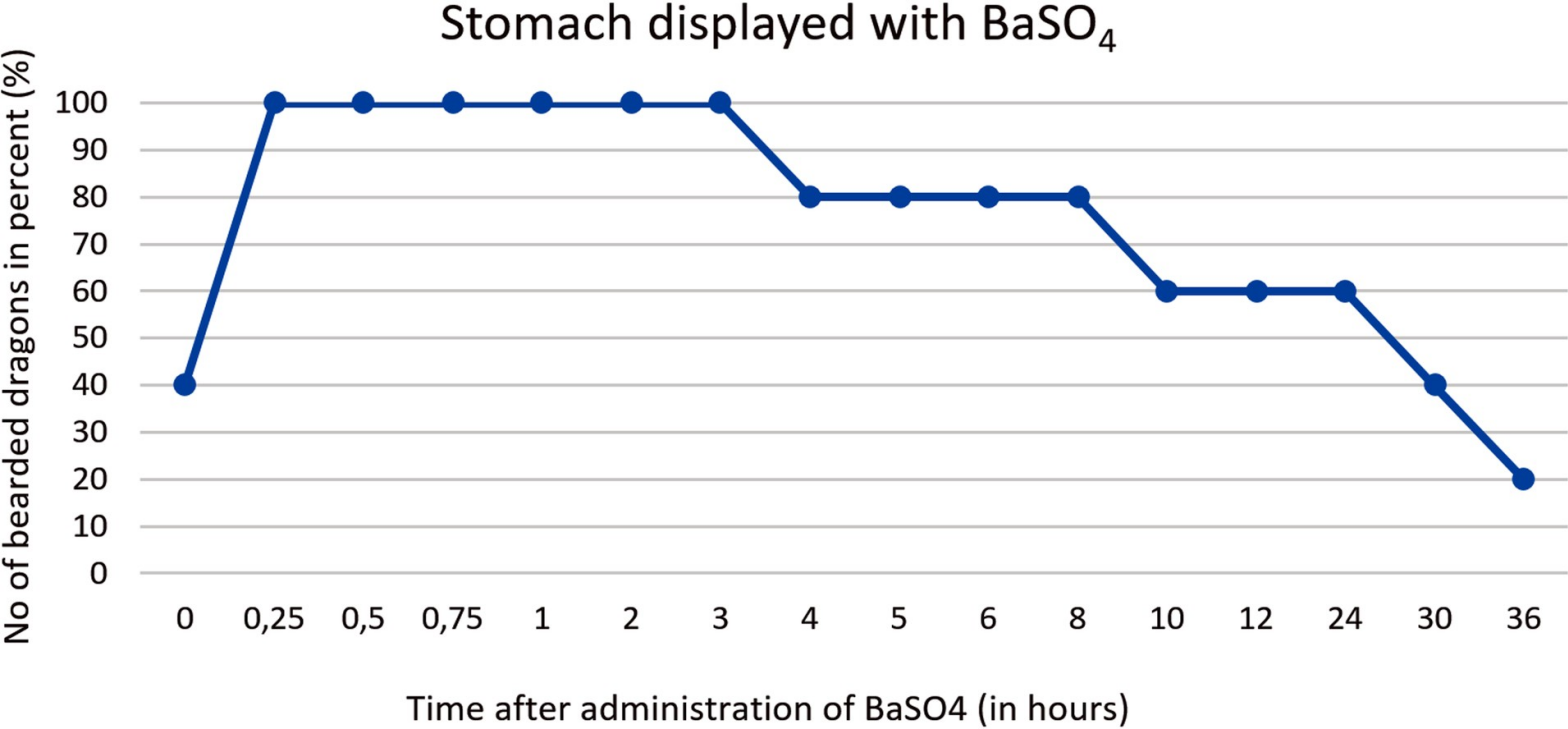
BaSO_4_ contrast visibility in the stomach over time. Percentage of animals and frequency at measuring time point; y = number of animals in percent (%) in which contrast media could be found in the stomach; x = time point during the BaSO_4_contrast passage (hours).

Contrast of the stomach could be demonstrated in all five animals within 2 hours (Tables 4 and 5, Figs 8 and 9). In the dorsoventral view, the stomach appeared as an elongated “C” and was primarily located on the left side of the coelomic cavity (Fig 8). Laterolateral radiographs showed a “cigar-shaped” stomach located in the ventral coelomic cavity (Fig 9). Longitudinal folds could be seen in the stomach with BaSO_4_ in four out of five animals. BaSO_4_ completely filled the stomach within two hours in all five animals, and complete emptying time was 4, 10, 30 and 36 hours and even more than 36 hours in one animal. This bearded dragon maintained BaSO_4_ in the stomach for the entire 36 hours study (Tables 4 and 5). In all five bearded dragons, stomach contrast lasted from 15 minutes to over 36 hours (Fig 10). Fig 10 illustrates the time that BaSO_4_ was found in the stomach of the five bearded dragons.

#### Intestinum tenue

**Fig 11:**
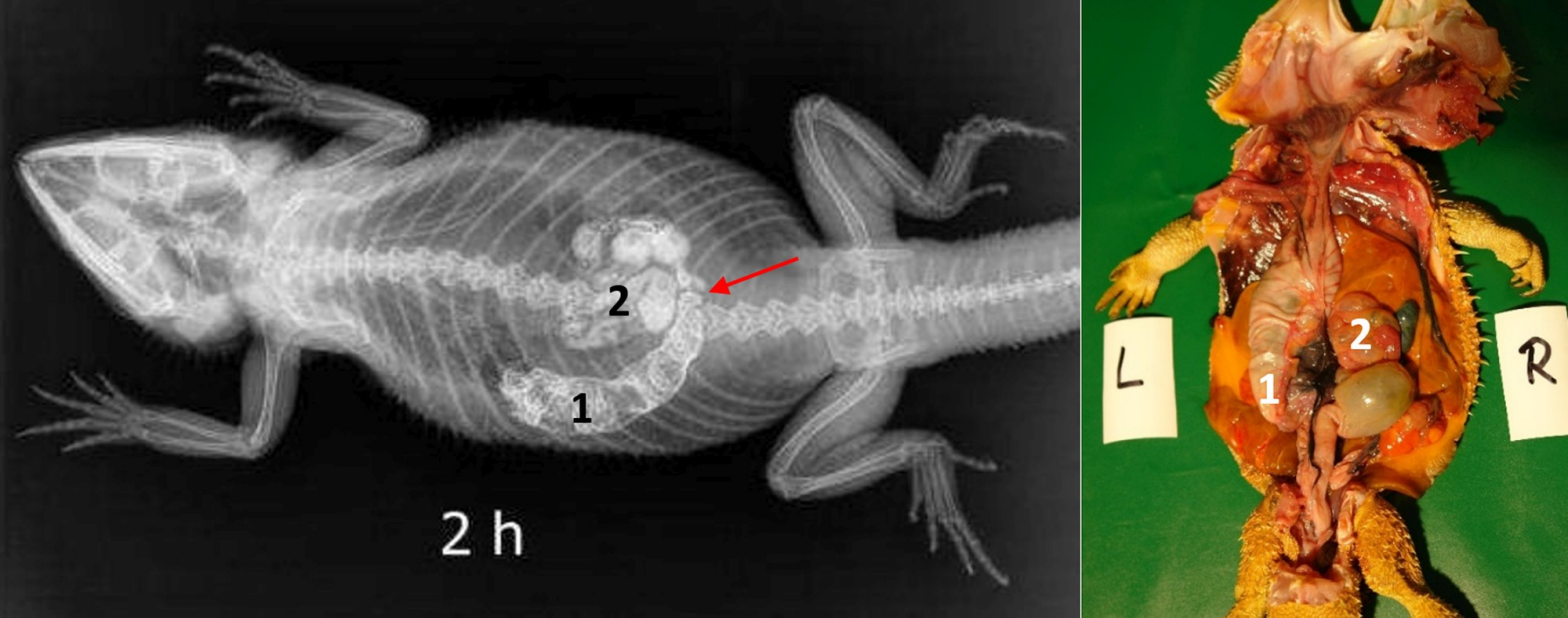
Dorsoventral (DV) radiograph 2 hours after oral administration of BaSO_4_. 2 hours after oral administration of BaSO_4_, (kV 42, mAs 2). The stomach (1) is illustrated by the presence of BaSO_4_; it is located on the left side of the coelomic cavity and is emptying from oral to aboral. The first loops of the small intestine (2) are displayed with BaSO_4_. Peristaltic movement can be observed in the small intestines (red arrow).

**Fig 12:**
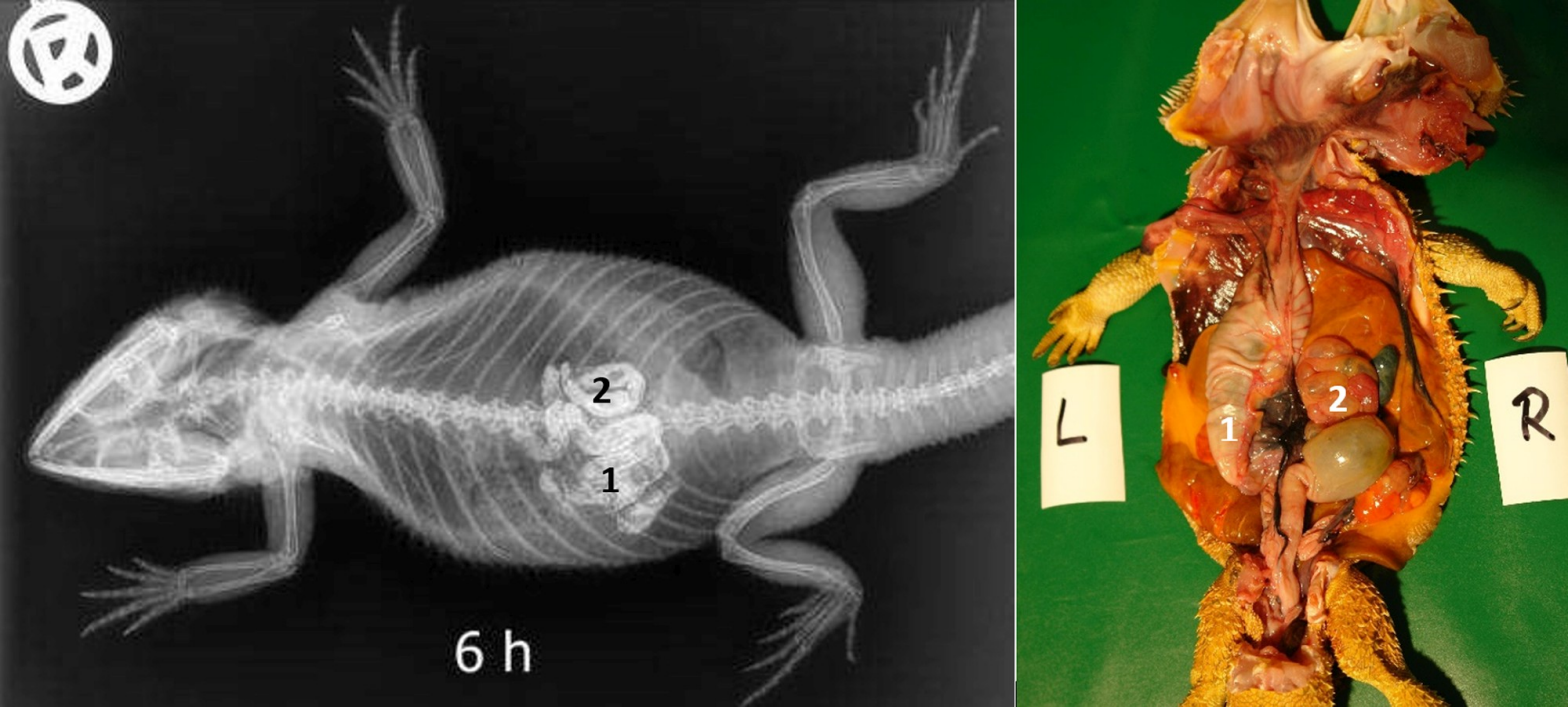
Dorsoventral (DV) radiograph 6 hours after oral administration of BaSO_4_. 6 hours after oral administration of BaSO_4_, (kV 42, mAs 2). Residual BaSO_4_ can be seen in the caudal (pyloric) stomach (1). Enhanced contrast of the loops of the small intestine (2) is observed at this time.

**Fig 13:**
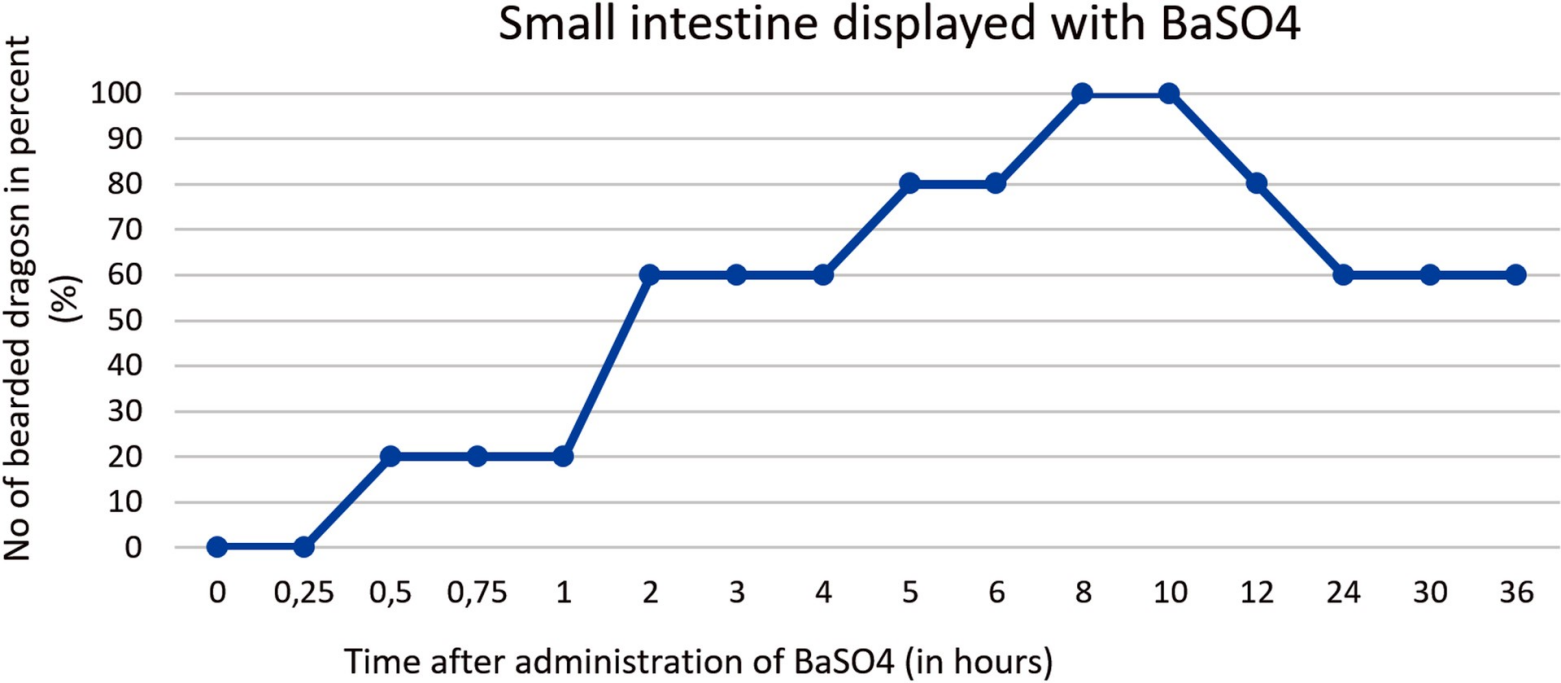
BaSO_4_ contrast visibility in the small intestines over time. Percentage of animals and frequency at measuring time point; y = number of animals in percent (%) in which contrast media could be found in the small intestine; x = time point during the BaSO_4_ contrast passage (hours).

There was a high degree of variance in the small intestine positioning within the coelomic cavity. Loops of the small intestine were primarily located on the left side of the coelomic cavity in all 5 bearded dragons; however, in one out of five cases the small intestines could be found on the midline and in one out of five cases on the right side of the caudal third of the coelom (Figs 11 and 12). Fig 13 shows the BaSO_4_passage time through the small intestines in all 5 bearded dragons. The filling of the small intestinal loops was variable in all five bearded dragons (Tables 4 and 5). To illustrate the small intestine with BaSO_4_ in all five bearded dragons, eight to ten hours was necessary (Fig 13).

#### Intestinum crissum

**Fig 14:**
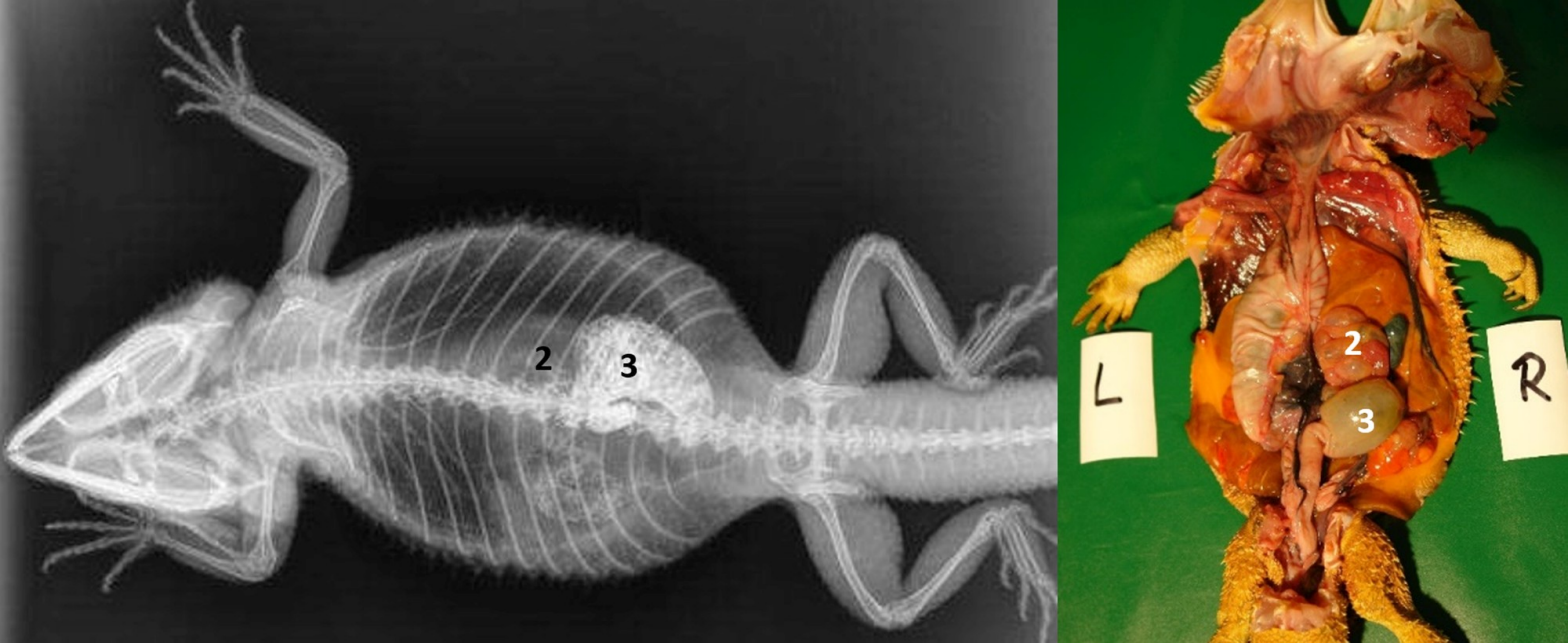
Dorsoventral (DV) radiograph 36 hours after oral administration of BaSO_4_. 36 hours after oral administration of BaSO_4_, (kV 42, mAs 2). Note residual amount of BaSO_4_ in the intestinal loops (2) at 36 hours. The ampulla coli (3) can be identified with BaSO_4_ on the right side of the coelomic cavity at the 10^th^ thoracic vertebrae and 2 ^nd^ lumbar vertebrae.

**Fig 15:**
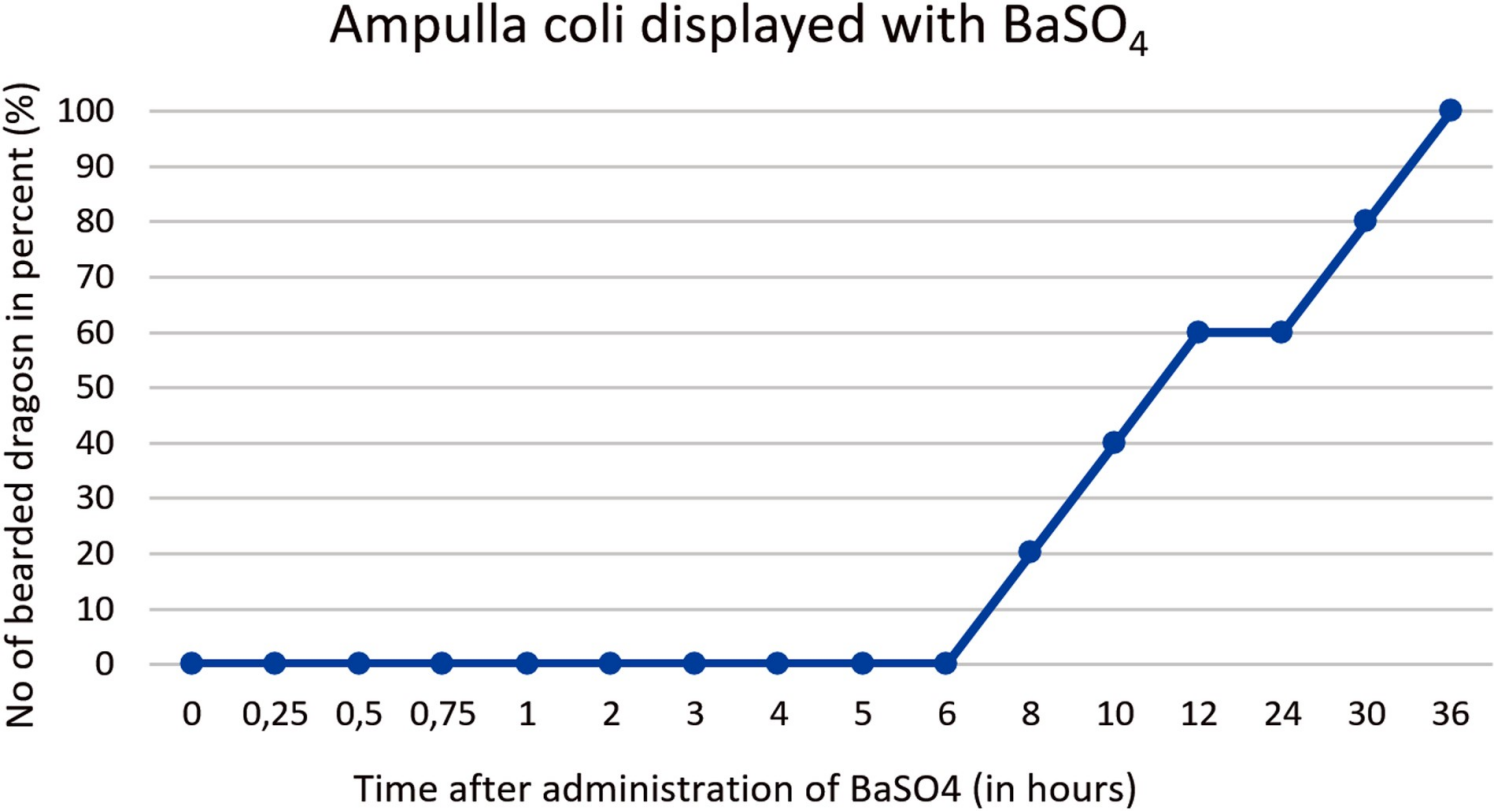
BaSO_4_ contrast visibility in the ampulla coli over time. Percentage of animals and frequency at measuring time point; y = number of animals in percent (%) in which contrast media could be found in the ampulla coli; x = time point during BaSO_4_ contrast passage (hours).

Ampulla coli. The ampulla coli is displayed as a sack-like enlargement and is located on the right side of the midline in the caudal coelom (Fig 14). Fig 15 illustrates the BaSO_4_ passage time through the ampulla coli in all five bearded dragons. The filling of the ampulla coli was variable in all five bearded dragons (Tables 4 and 5). To illustrate the ampulla coli with BaSO_4_ in all five bearded dragons, 30 to 36 hours was necessary (Fig 15).

**Fig 16:**
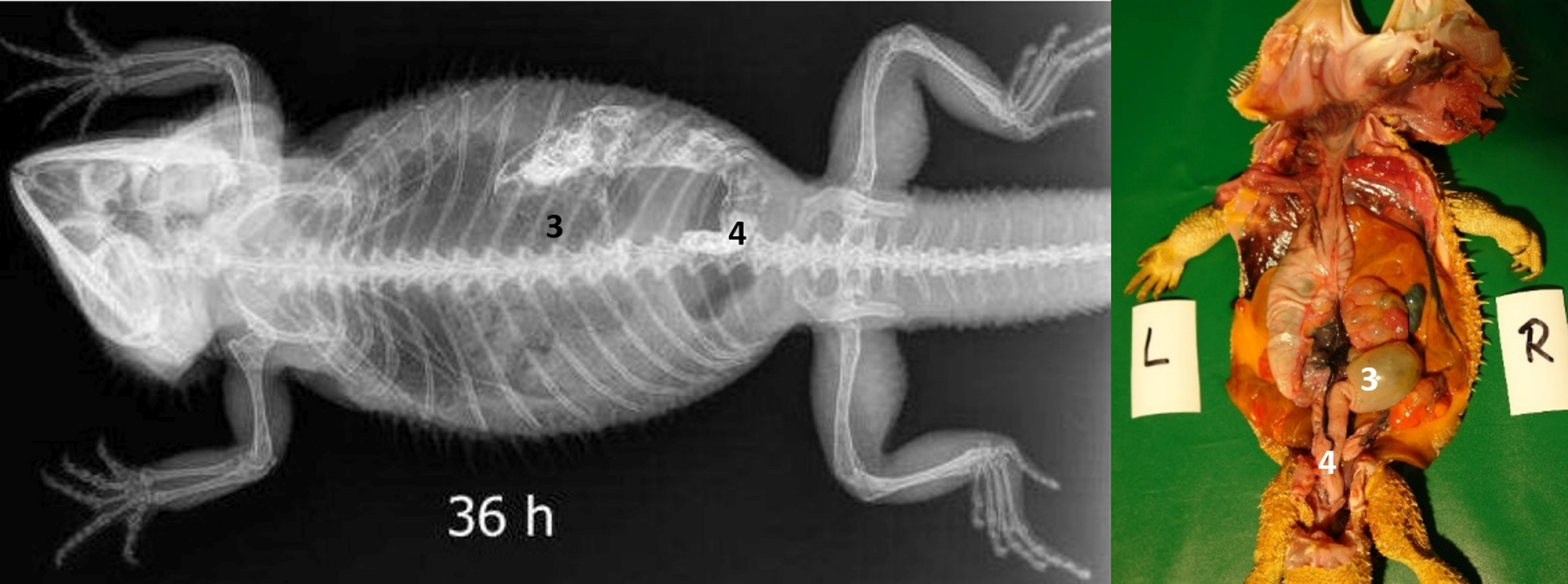
Dorsoventral (DV) radiograph 36 hours after oral administration of BaSO_4_. 36 hours after oral administration of BaSO_4_, (kV 42, mAs 2). The BaSO_4_ has passed through the ampulla coli (3) and into the rectum (4), which has an “U-shape” and is located on the right side of the coelomic cavity running parallel to the spinal column.

**Fig 17:**
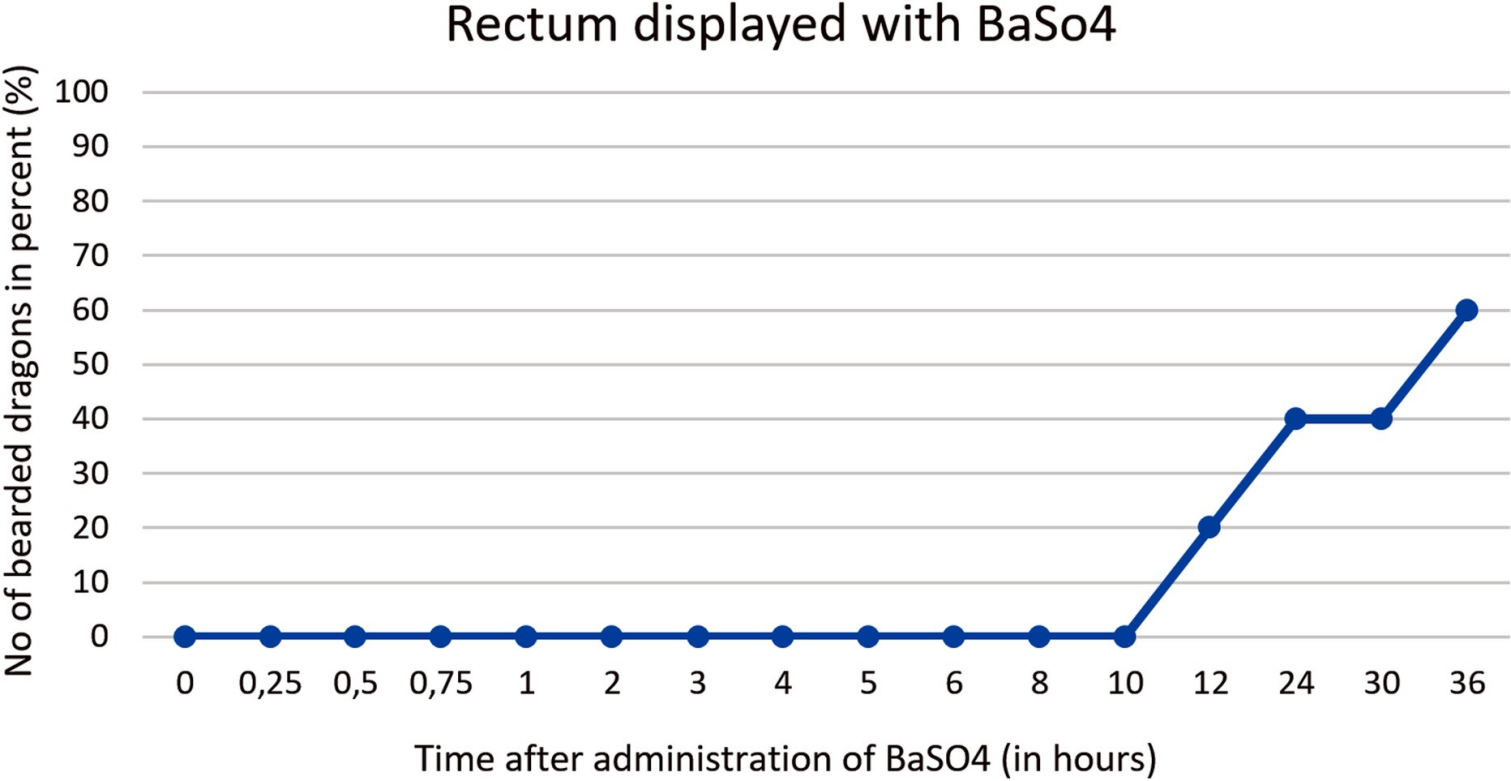
BaSO_4_ contrast visibility in the rectum over time. Percentage of animals and frequency at measuring time point; y = number of animals in percent (%) in which contrast media could be found in the rectum; x = time point during BaSO_4_contrast passage (hours).

Rectum. The rectum is illustrated as an “U-shape” (OR as an U) and is located on the right side of the coelomic cavity running parallel to the vertebral column (Fig 16). The filling of the rectum was variable in all five bearded dragons (Tables 4 and 5). Fig 17 illustrates BaSO_4_ passage time through the rectum in all 5 bearded dragons. 36 hours of passage time was not sufficient to illustrate the rectum with BaSO_4_ in all five bearded dragons (Fig 17).

Cloaca. BaSO_4_ was not observed in any of the bearded dragon cloaca’s after 36 hours (Tables 4 and 5).

### Sodium/Meglumine–Amidotrizoate (SMAT; Gastrografin) contrast media studies

Sex, bodyweight, SVL and age of the 21 bearded dragons (12 females, 9 males) with oral administration of SMAT (Gastrografin) are listed below in Table 6.

**Table 6:** Descriptive statistics for the adult bearded dragons used in the Sodium/Meglumine–Amidotrizoate (SMAT, Gastrografin) contrast media study.

Number	Sex	Bodyweight (in g)	Snout-vent length (in cm)	Animal age (in years)
1	female	256	19.2	adult
2	female	272	20.5	adult
3	male	250	19.0	2
4	female	264	19.3	2
5	female	327	22.7	adult
6	male	356	22.5	adult
7	female	309	18.6	9
8	male	373	20.6	9
9	female	286	19.9	9
10	male	547	22.6	7
11	female	216	17.9	>3
12	male	172	18.5	>3
13	male	146	16.0	2
14	female	109	16.1	3
15	female	101	15.1	3
16	male	127	17.5	3
17	female	220	16.6	3
18	male	359	18.7	3
19	female	177	17.2	2
20	male	366	19.4	>9
21	female	364	19.1	>6

Animal number, gender, bodyweight (g), snout-vent length (SVL) (cm) and age (years) of bearded dragons administered Sodium/Meglumine–Amidotrizoate (SMAT, Gastrografin).

SVL = snout-vent length

SMAT (Gastrografin), was delivered at 5 ml/kg by syringe per os and ingested willingly by all of the bearded dragons.

Table 7 gives an overview of the course of the contrast study with SMAT (Gastrografin), including time and location within the digestive tract in all 21 bearded dragons.

**Table 7:** Transit times for SMAT contrast in bearded dragons.

Contrast medium:	Location	Min	Max	Median
**(SMAT) Gastrografin**	**Oropharynx** (L 2)	**0**	**0**	**0**
	**Oropharynx** (L 5)	**0.25**	**4**	**0.25**
**(SMAT) Gastrografin**	**Esophagus** (L 2)	**0**	**< 0.5**	**0–0.25**
	**Esophagus** (L 5)	**0.25**	**1**	**0.25**
**(SMAT) Gastrografin**	**Stomach** (L 2)	**0**	**0.5**	**0.25**
	**Stomach** (L 5)	**5**	**36**	**24**
**(SMAT) Gastrografin**	**Small intestine** (L 2)	**0.25**	**6**	**2**
	**Small intestine** (L 5)	**10**	**>36**	**24**
**(SMAT) Gastrografin**	**Ampulla coli** (L 2)	**4**	**10**	**8**
	**Ampulla coli** (L 5)	**>36**	**>36**	**>36**
**(SMAT) Gastrografin**	**Rectum** (L 2)	**6**	**>36**	**10**
	**Rectum** (L 5)	**30**	**>36**	**>36**
**(SMAT) Gastrografin**	**Cloaca** (L 2)	**>36**	**>36**	**>36**
	**Cloaca** (L 5)	**>36**	**>36**	**>36**

The table is displaying the type of contrast medium (Sodium/Meglumine–Amidotrizoate; SMAT; Gastrografin); location within the digestive tract; and the minimum (Min), maximum (Max) and median (Median) times (hours), sections of the digestive tract were completely filled (L2 = Level 2, top row) and completely emptied (L5 = Level 5, bottom row). Min = minimum time, Max = maximum time; Median = median time.

#### Oropharynx and esophagus

**Fig 18:**
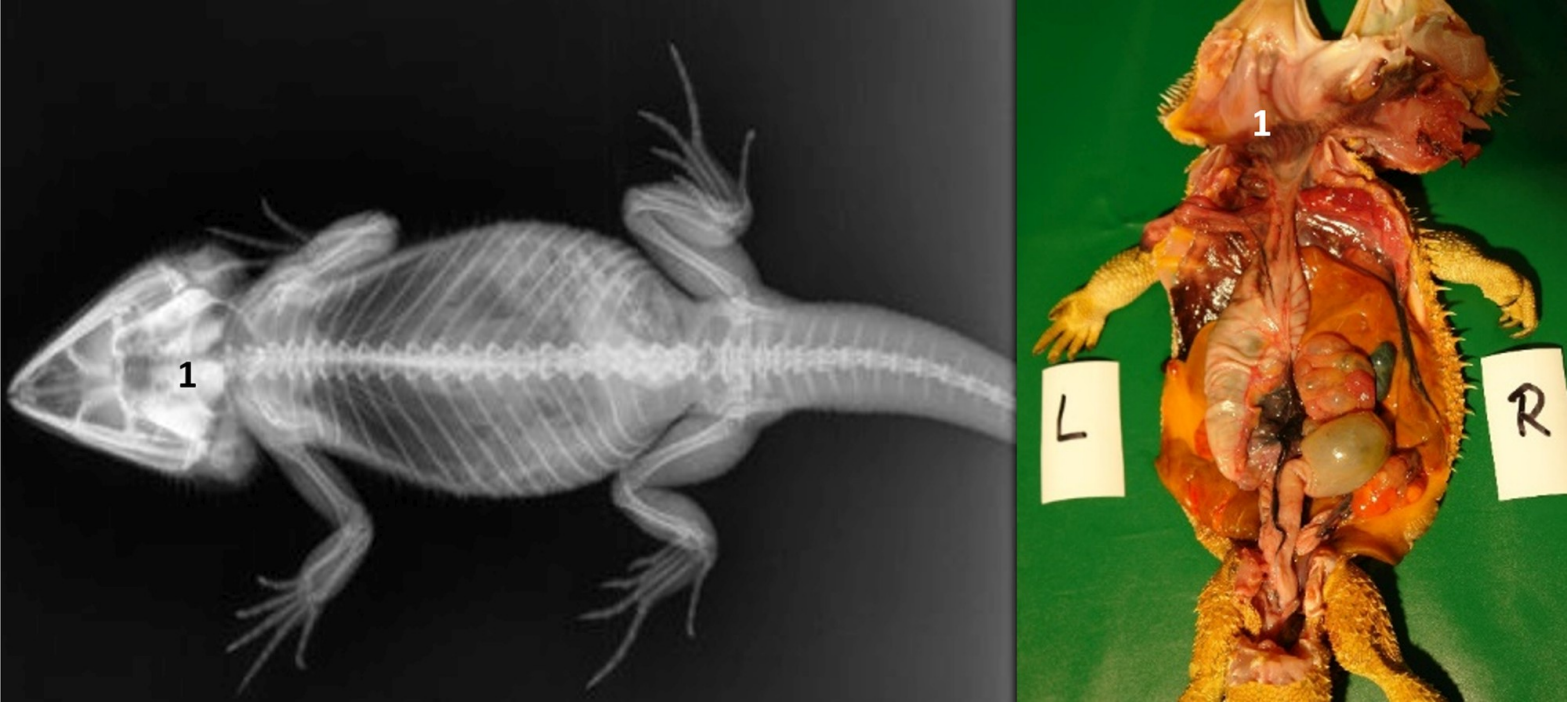
Dorsoventral (DV) radiograph immediately after oral administration of SMAT. Immediately after oral administration of SMAT (kV 42, mAs 2). Contrast medium can be seen in the oropharynx (1).

**Fig 19:**
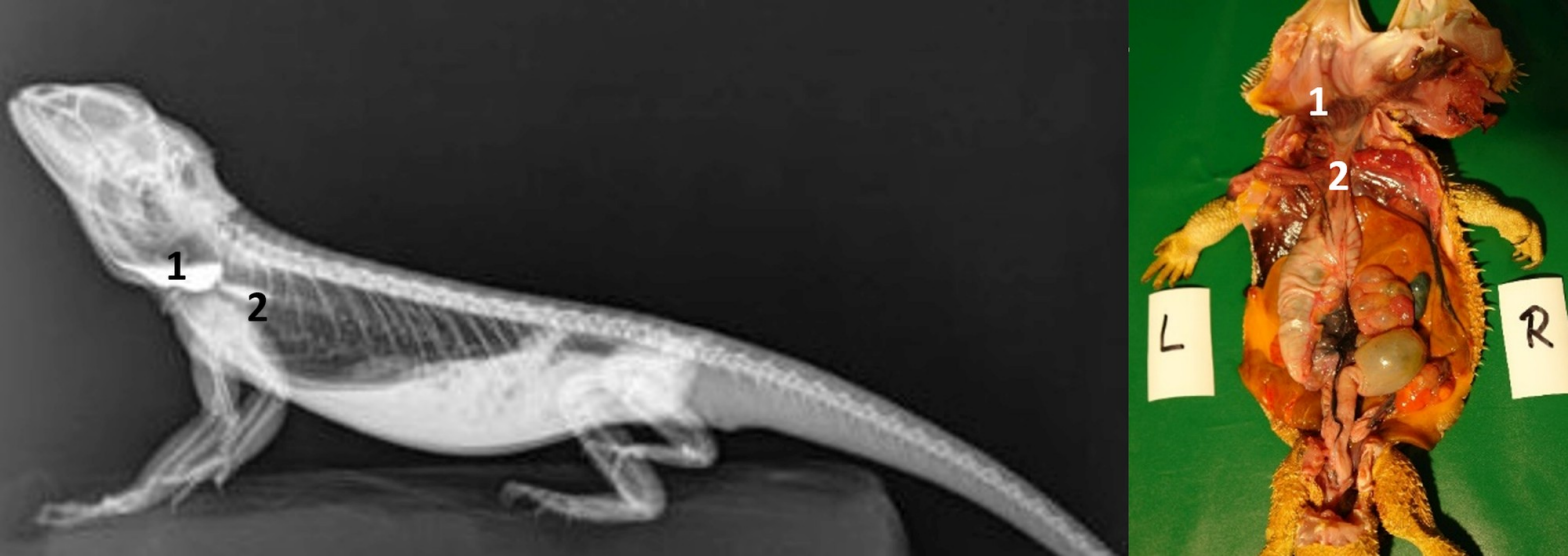
Laterolateral (LL) radiograph in sternal recumbency using a horizontal beam immediately after oral administration of SMAT. Immediately after oral administration of SMAT (kV 42, mAs 2). Contrast medium is visible in the oropharynx (1), with a small volume also seen entering the esophagus (2).

**Fig 20:**
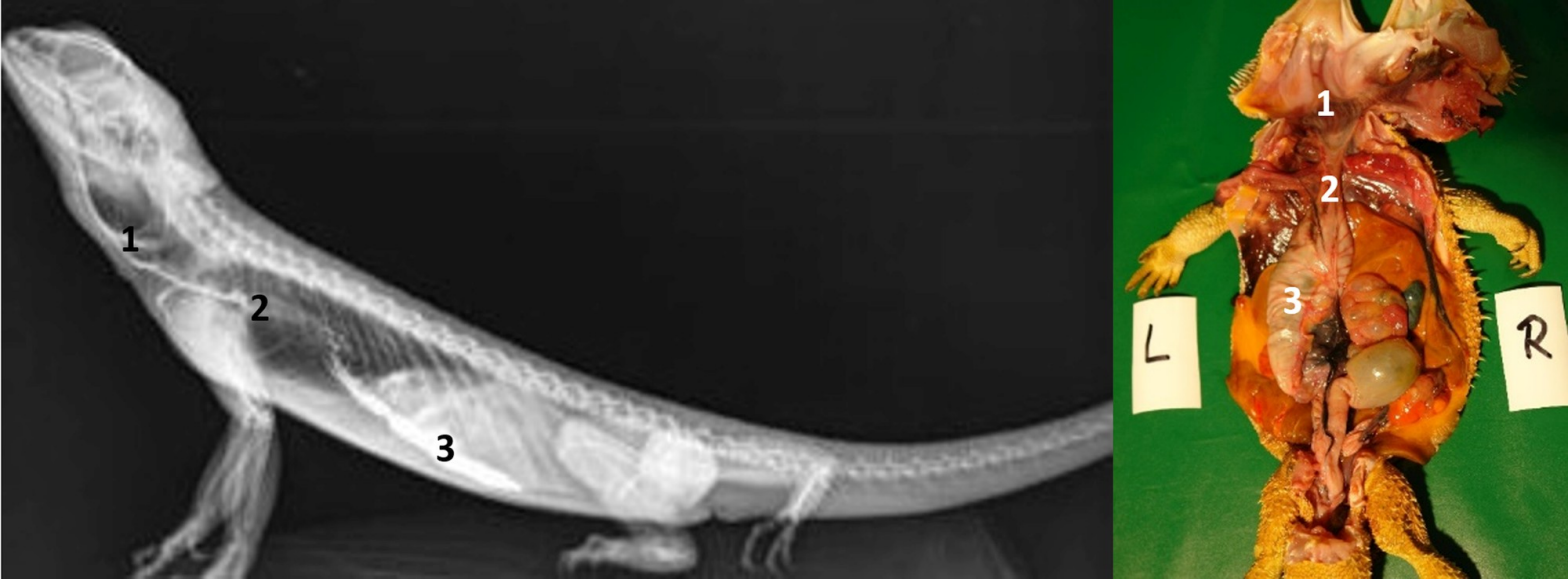
Laterolateral (LL) radiograph in sternal position using a horizontal beam immediately after oral administration of SMAT. Immediately after oral administration of SMAT (kV 42, mAs 2). Contrast medium illustrates oropharynx (1), esophagus (2), and stomach (3).

**Fig 21:**
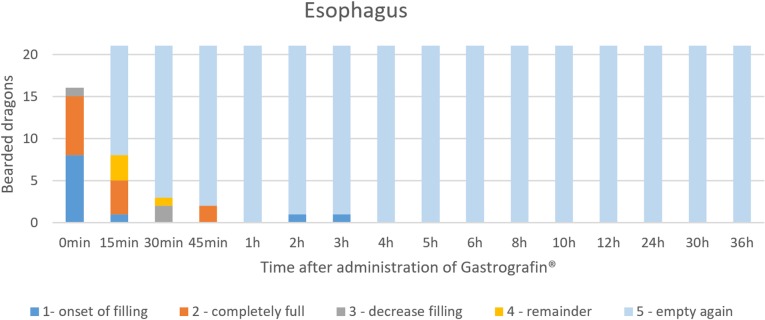
SMAT contrast visibility in the esophagus over time. Frequency at measuring time point; y = number of animals in which contrast media could be found in the esophagus; x = time point during the Sodium/Meglumine–Amidotrizoate (SMAT); (Gastrografin) contrast passage (in minutes and hours).

Directly after the administration of SMAT (Gastrografin), the oropharynx (Fig 18) was visible in all 21 animals (100%). In the lateral radiograph, display of contrast medium in the esophagus was possible within 15 min (Table 7, Figs 19, 20 and 21). Display of the esophagus in the dorsoventral view was difficult because of the vertebral column. Fig 21 illustrates the SMAT (Gastrografin) passage time through the esophagus in all 21 bearded dragons. To illustrate the esophagus with SMAT in all 21 animals, immediately (0 minutes) and up to 15 minutes after application was a good time set (Fig 21).

#### Stomach

**Fig 22:**
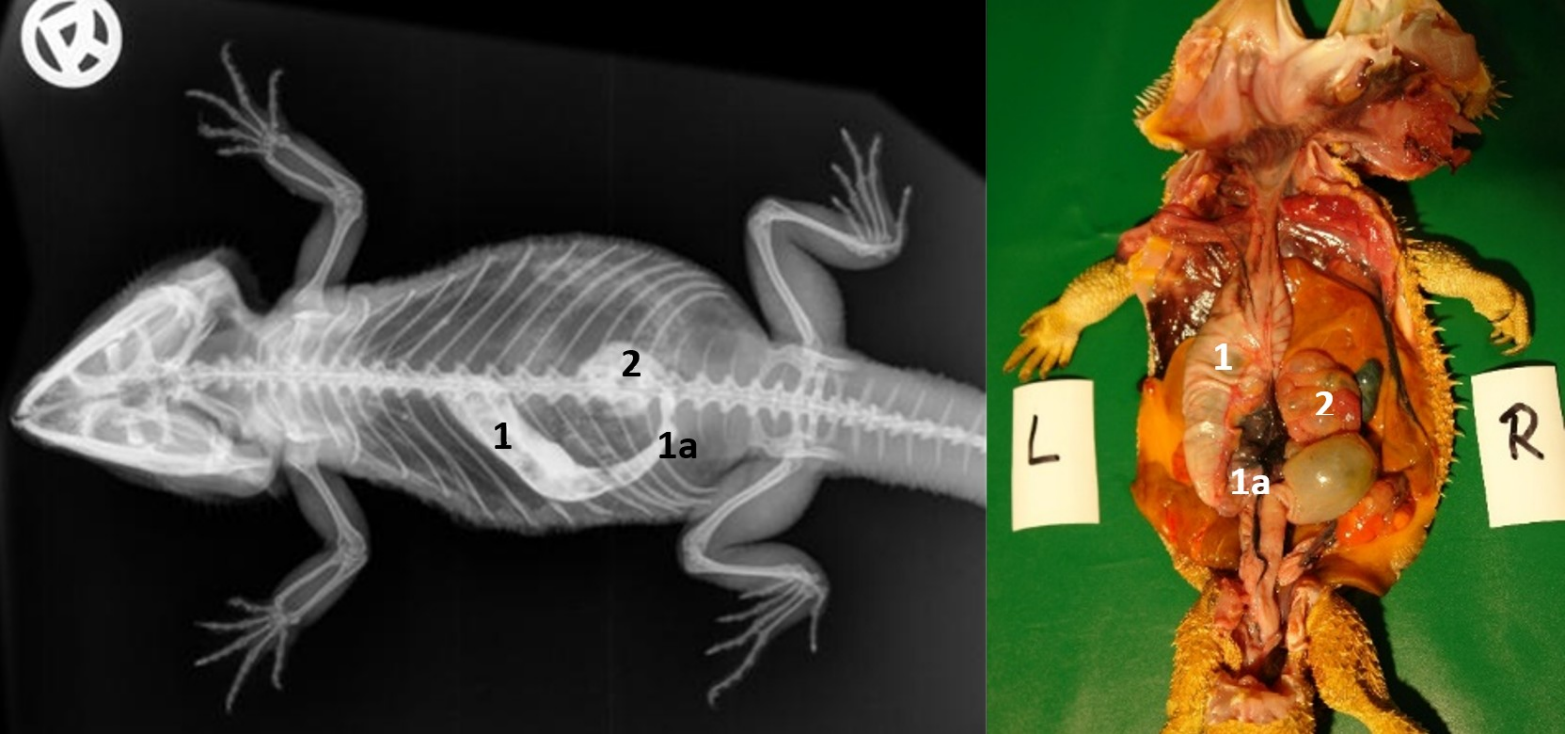
Dorsoventral (DV) radiograph 15 minutes after oral administration of SMAT. 15 minutes after oral administration of SMAT (kV 42, mAs 2). The stomach (1, 1a) and cranial section of the small intestine (2) are highlighted with contrast medium. The stomach is visible on the left side of the coelom and the cardia is located between the 6^th^ and 7^th^ thoracic vertebrae. The pylorus is visible at the first lumbar vertebrae (1a) and some contrast medium can be seen entering the small intestine (2).

**Fig 23:**
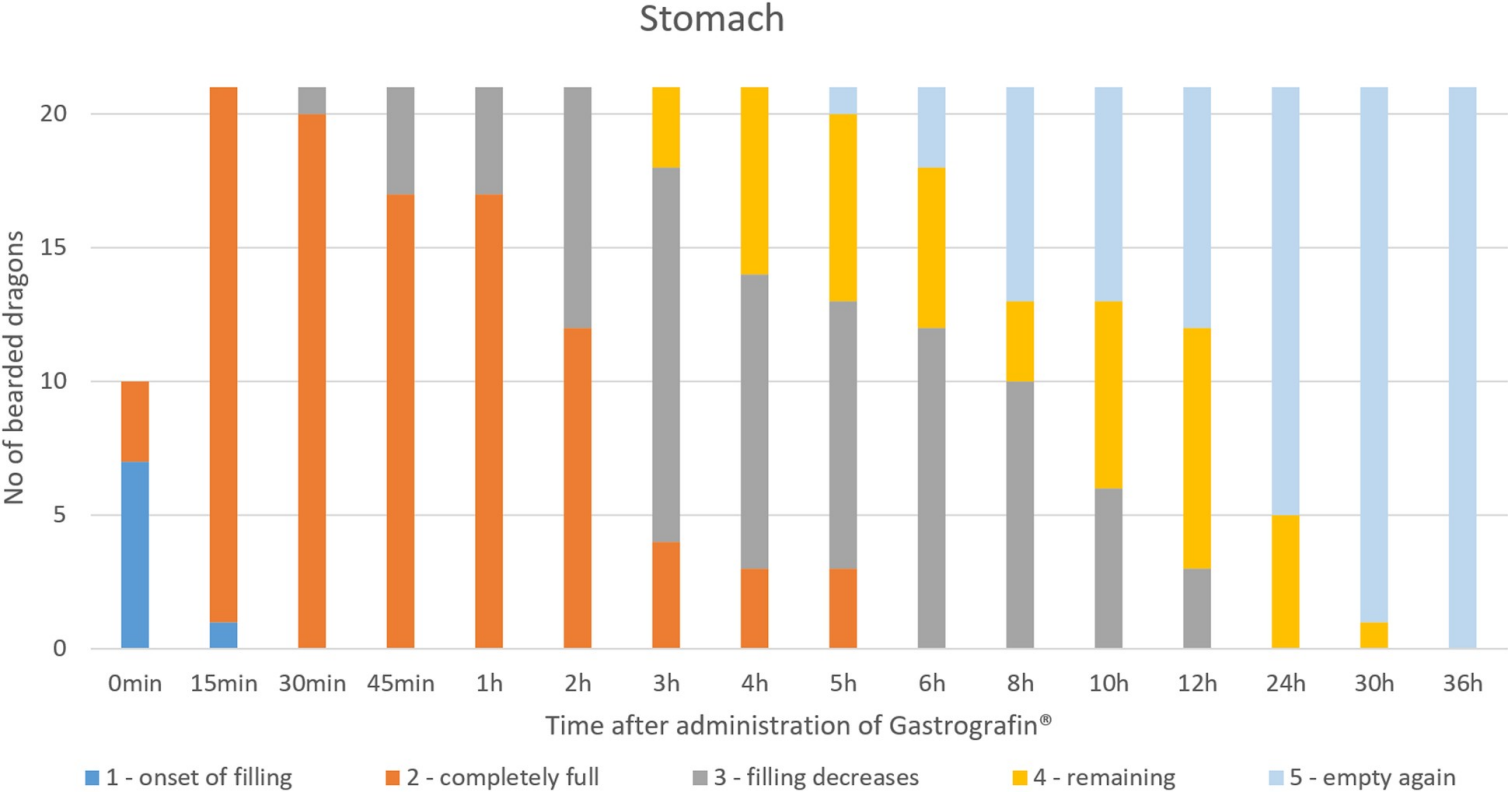
SMAT contrast visibility in the stomach over time. Frequency at measuring time point; y = number of animals in which contrast media could be found in the stomach; x = time point during the SMAT; (Gastrografin) contrast passage (in minutes and hours).

**Fig 24:**
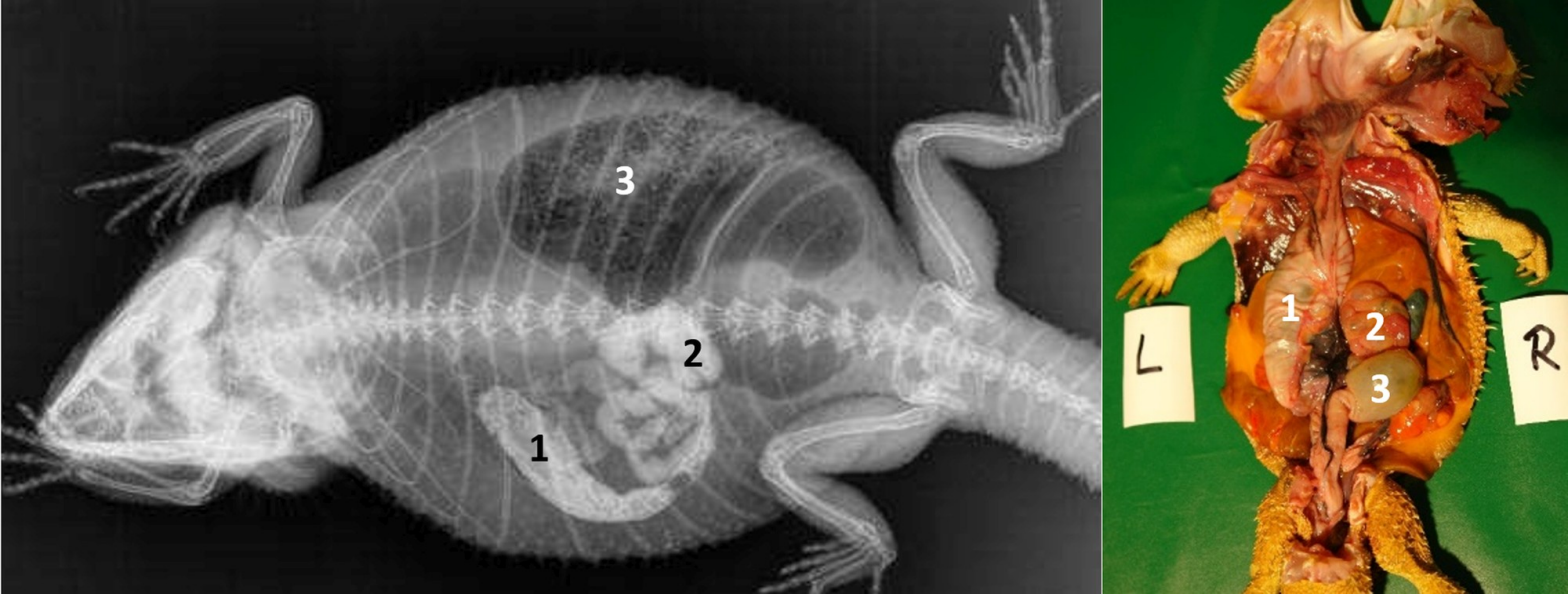
Dorsoventral (DV) radiograph 3 hours after oral administration of SMAT. 3 hours after oral administration of SMAT (kV 42, mAs 2). The stomach (1) is visible on the left side of the coelom and transition to the small intestine (2) occurs laterally between the 9^th^ and 10^th^ ribs. The ampulla coli (3) contains gas and sand particles.

SMAT could be seen in the stomach within 30 minutes of dosing (Table 7, Figs 22 and 23). In the dorsoventral view, the stomach was elongated, “C-shaped”, and primarily on the left side of the coelom (Figs 22 and 24) between the 4^th^ thoracic vertebra (cardia) and the 3^rd^ lumbar vertebra (pyloric region). Mucosal folds and peristalsis were apparent and the stomach contents clearly visible with SMAT (Gastrografin). Fig 23 illustrates the SMAT (Gastrografin) passage time through the stomach in all 21 bearded dragons. To visualize the stomach with SMAT, 15 to 30 minutes post-dosing is considered ideal (Fig 23). The stomach was completely emptied of SMAT in all 21 lizards 36 hours post-dosing.

#### Intestinum tenue

**Fig 25:**
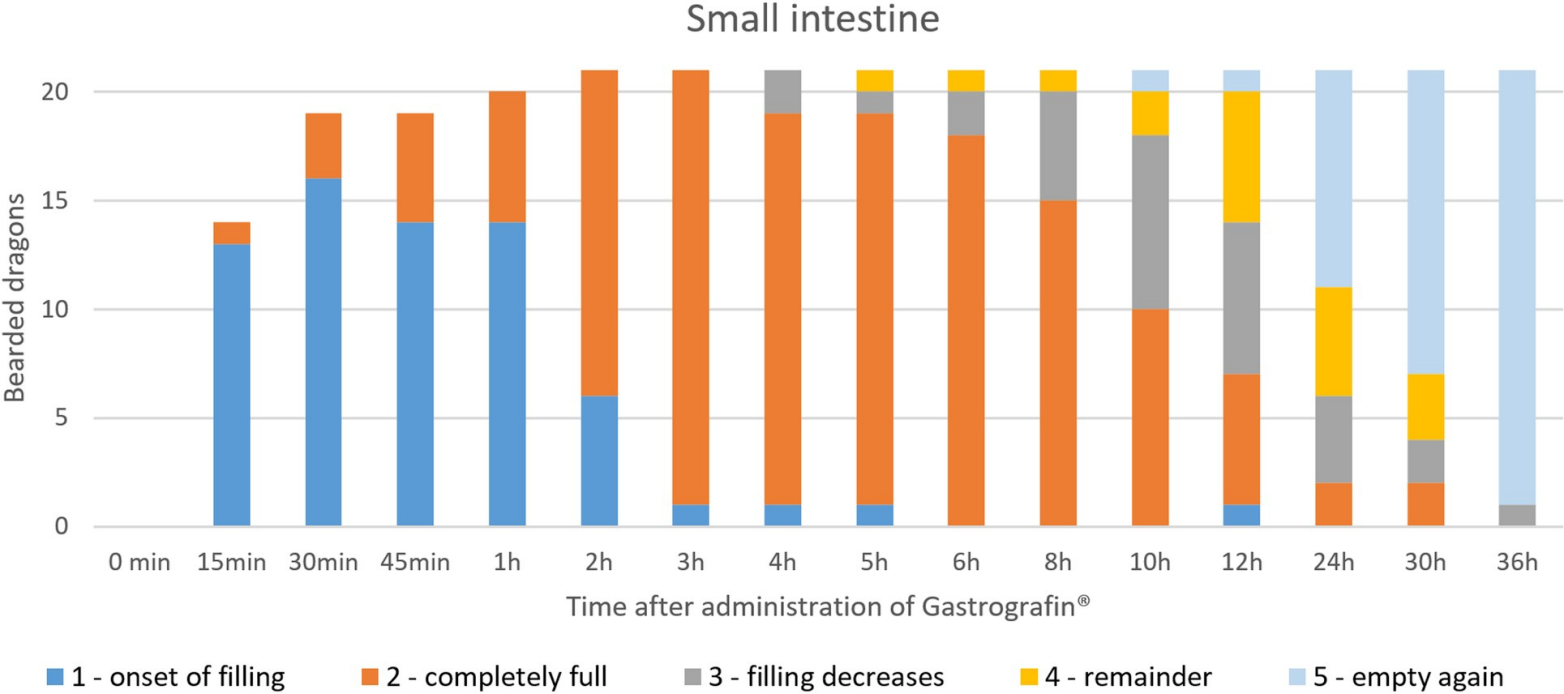
SMAT contrast visibility in the small intestine over time. Frequency at measuring time point; y = number of animals in which contrast media could be found in the small intestine; x = time point during the Sodium/Meglumine–Amidotrizoate (SMAT); contrast passage (in minutes and hours).

**Fig 26:**
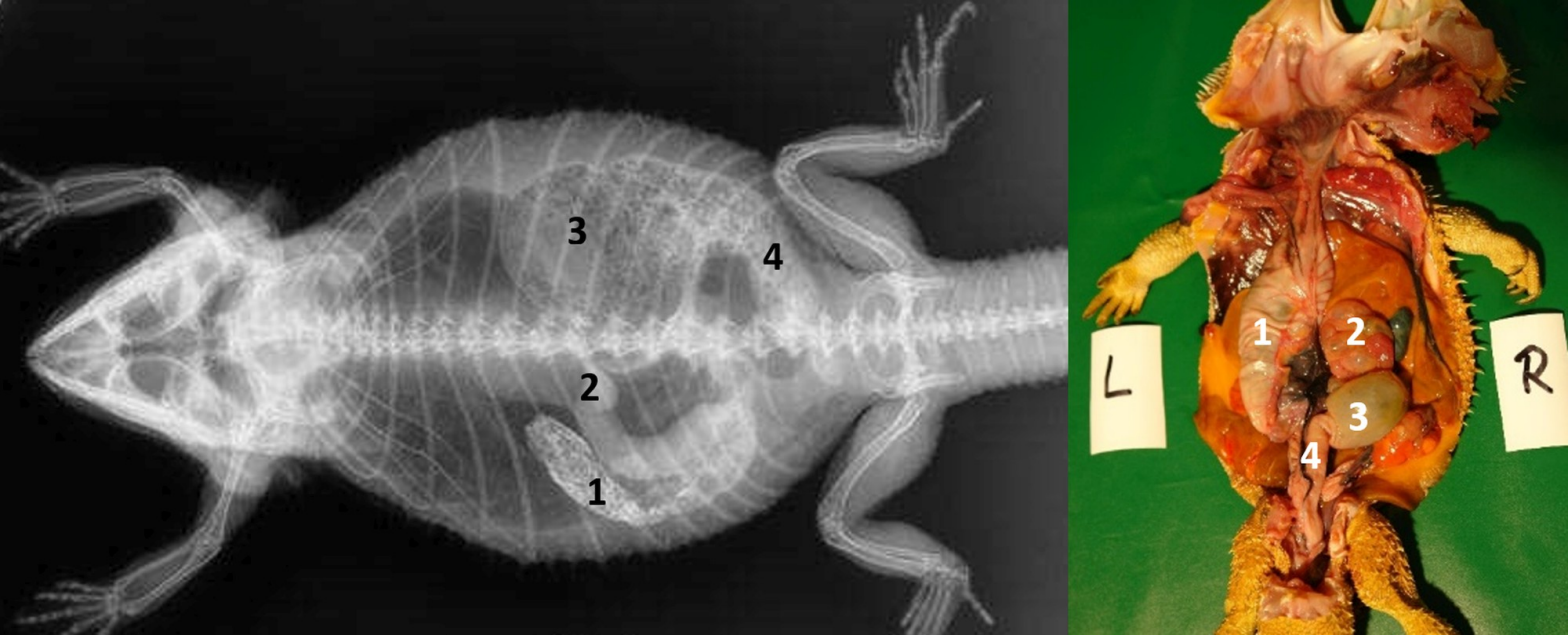
Dorsoventral (DV) radiograph 12 hours after oral administration of SMAT. 12 hours after oral administration of SMAT (kV 42, mAs 2). The caudal aspect of the stomach (pylorus) (1) is filled with residual contrast; the small intestine (2) can be seen merging into the ampulla coli (3) at the level of the 9 ^th^ thoracic vertebrae; and the rectum (4) can be seen filling with contrast.

SMAT illustrated the small intestines (Figs 22, 24, 25, 26 and 15) in all 21 animals (100%) (Table 7, Fig 25). In the dorsoventral views, illustration of the small intestine was possible within 6 hours (Table 7, Figs 24 and 25). In the laterolateral views, individual bowel segments were not illustrated very well due to soft tissue superimposition; however, dorsoventral views were useful in revealing the different details of the small intestine. The small intestine´s intracoelomic position was variable in all 21 bearded dragons (Figs 22, 24, 26 and 15); however, they could be found most commonly in the left caudal half of the coelom (Figs 22, 24 and 26). In some animals, pronounced fat bodies and ovaries or eggs in the salpinx did cause displacement of the small intestines into the cranial half of the coelom. Fig 25 summarizes the SMAT contrast passage time through the small intestine in all 21 bearded dragons. To illustrate the small intestines in bearded dragons with SMAT, radiographic images 3 to 6 hours post-dosing should be sufficient (Fig 25). In all but one bearded dragon, the small intestines were completely emptied of SMAT 36 hours post-dosing (Fig 25).

#### Intestinum crassum

**Fig 27:**
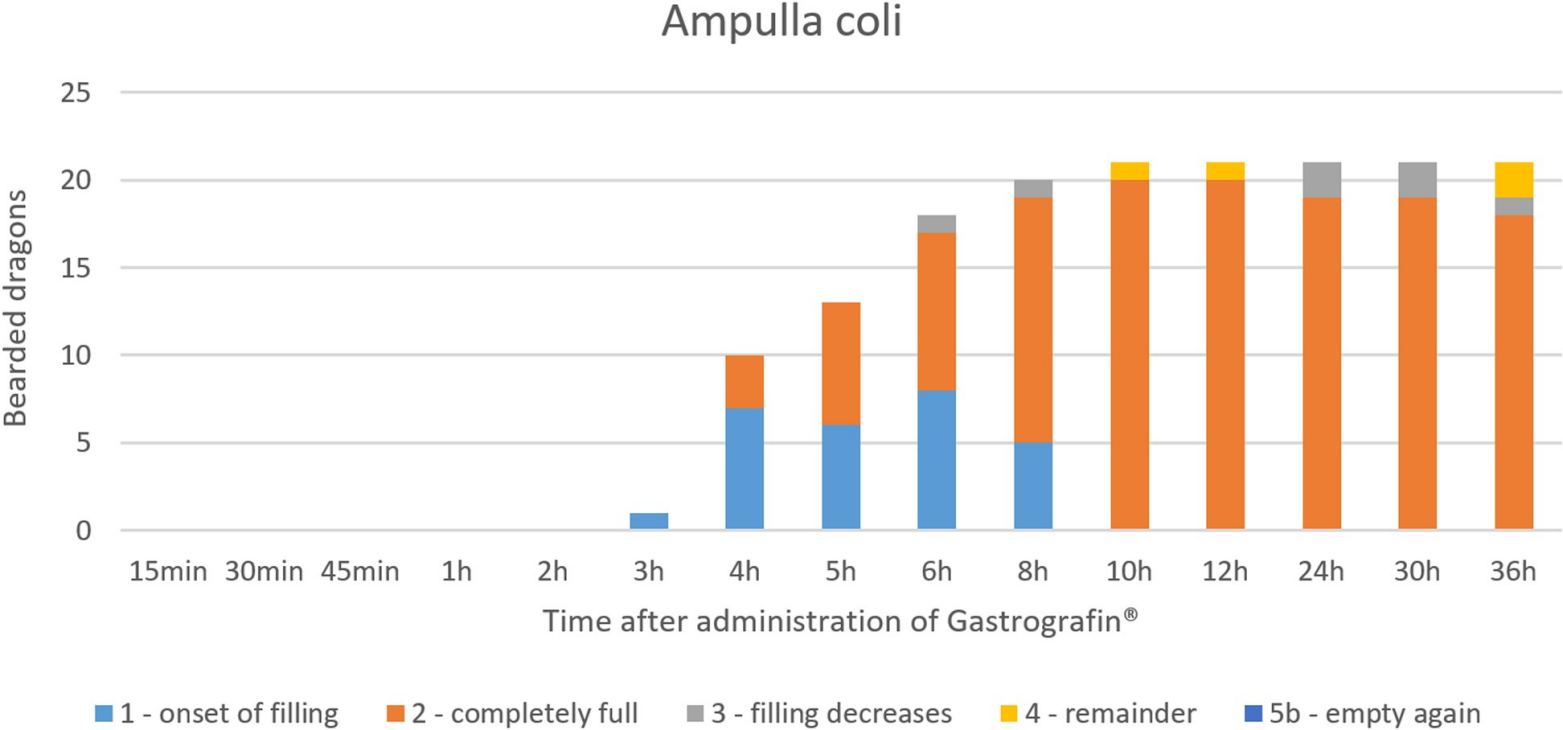
SMAT contrast visibility in the ampulla coli over time. Frequency at measuring time point; y = number of animals in which contrast media could be found in the ampulla coli; x = time point during the Sodium/Meglumine–Amidotrizoate (SMAT); contrast passage (in minutes and hours).

**Fig 28:**
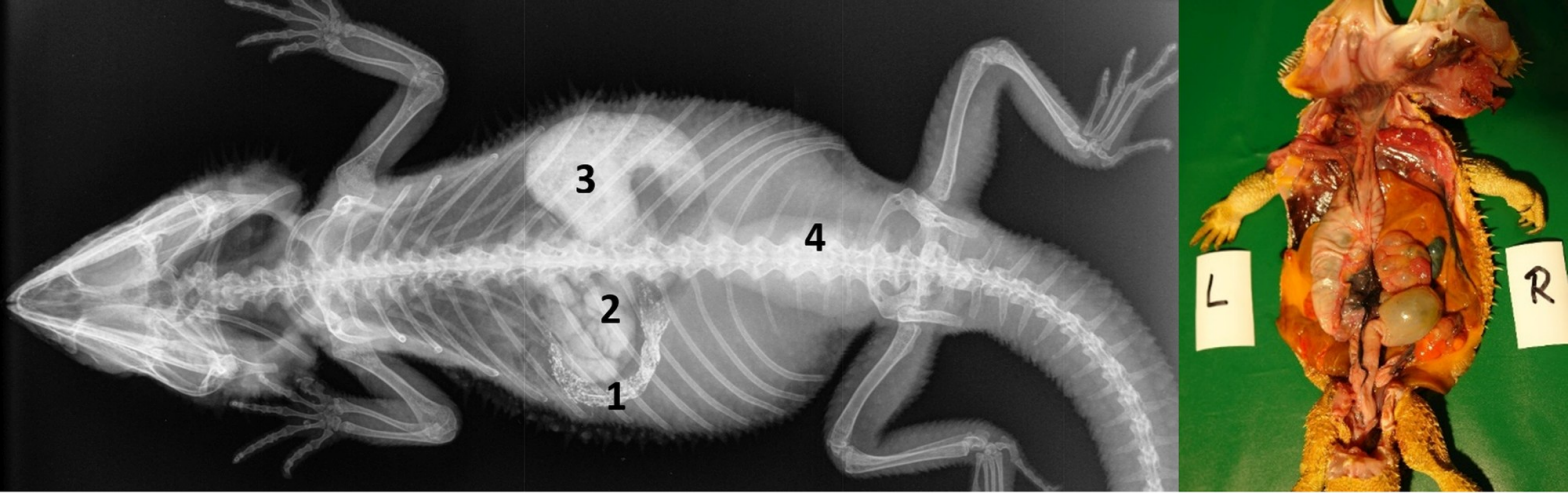
Dorsoventral (DV) radiograph 12 hours after oral administration of SMAT. 12 hours after oral administration of SMAT (kV 42, mAs 2). The stomach (1) and small intestine (2) are shifted cranially because of the prominent fat bodies. The ampulla coli (3) can be seen merging into the rectum (4), which has a width of three vertebral bodies and is far wider than the loops of the small intestine (2).

The ampulla coli (Figs 26, 27, 28 and 15) was visible in all 21 animals (100%) in the dorsoventral view (Table 7, Fig 27). The colic ampulla, which narrowed into the colic isthmus, had a spherical shape and was located in the caudal coelom on the right-side of midline (Figs 26, 28 and 15). In some bearded dragons, and before the SMAT reached the area, the ampulla coli was evident because of its mixture of gas and sand contents (Fig 24). The ampulla coli was fully illustrated 10 hours post SMAT dosing, and remained illustrated up to 36 hours in all of the bearded dragons (Fig 27).

**Fig 29:**
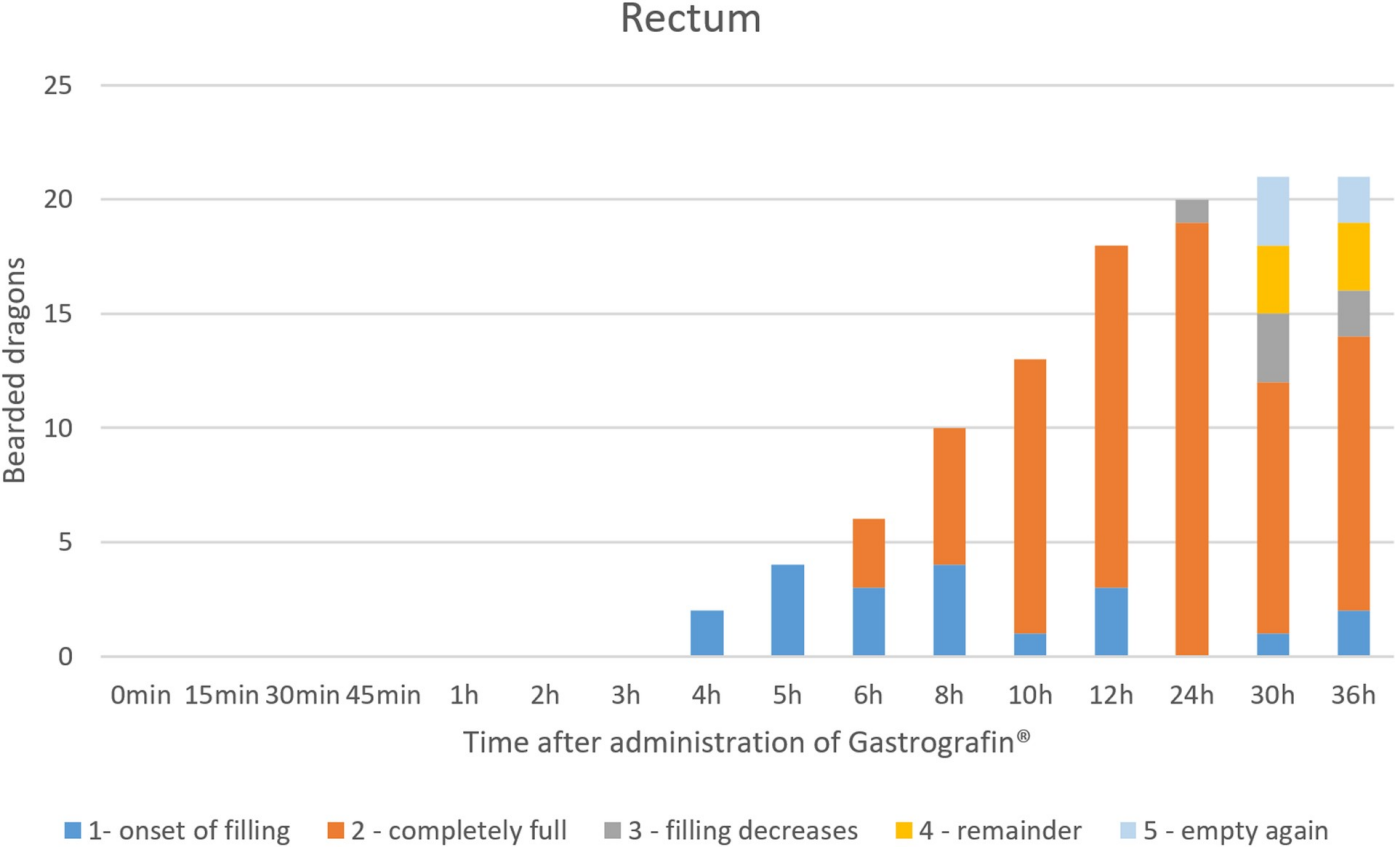
SMAT contrast visibility in the rectum over time. Frequency at measuring time point; y = number of animals in which contrast media could be found in the rectum; x = time point during the Sodium/Meglumine–Amidotrizoate (SMAT); contrast passage (in minutes and hours).

Rectum: In the dorsoventral view, SMAT was used to illustrate the rectum (Figs 26, 28 and 15) in all 21 animals (100%) (Table 7, Fig 29). The rectum varied in size (one to six vertebral body lengths), and was found in the lower right caudal quadrant of the coelom (Figs 26, 28 and 15). The rectum was best illustrated with SMAT in all 21 animals 12 to 24 hours post-dosing (Fig 29). SMAT was found in the rectum of all 21 dragons 36 hours post-dosing (Fig 29).

**Fig 30:**
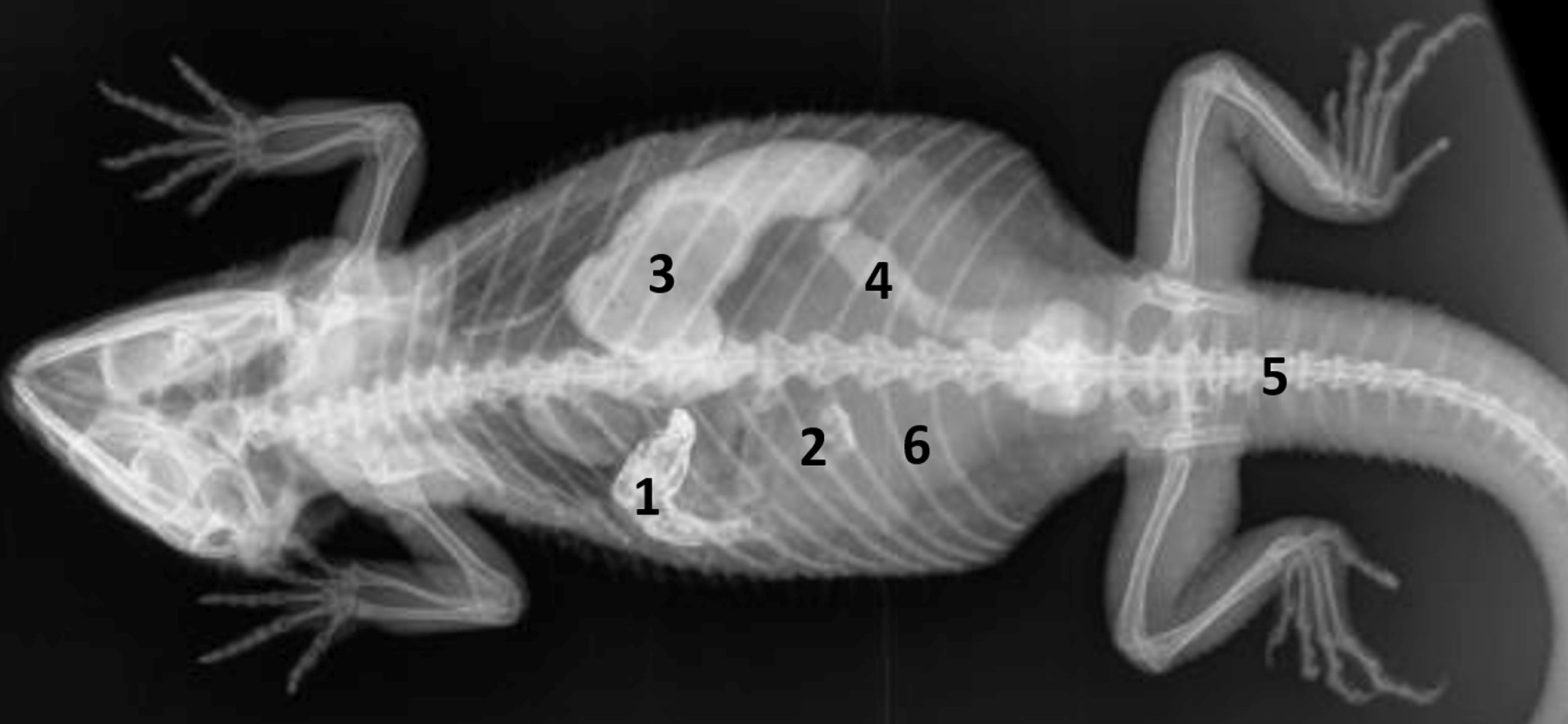
Dorsoventral (DV) radiograph 12 hours after oral administration of SMAT. 12 hours after oral administration of SMAT (kV 42, mAs 2). Prominent ovarian follicles (6) can be seen displacing the stomach (1) and small intestines cranially (2), both of which are filled with residual contrast. The ampulla coli (3) is filled with gas and contrast medium, while the rectum (4) can be seen on the right side of the coelom before emptying into the cloaca (5).

Pronounced fat bodies (Fig 28) and ovaries (Fig 30) or eggs in the salpinx could cause displacement of the small intestines into the cranial half of the coelom.

The cloaca was only visible when filled with contrast medium immediately before or during defecation (Table 7).

### Comparison of contrast studies using BaSO_4_ to SMAT

Comparison of the two contrast media for Level 2 is listed in Table 8 (new) as p- values of time point 1 (pTP1), p-value of time point 2 (pTP2) and p-value of duration of retention period time as p(TP2-TP1).

**Table 8:** Comparisons of the times to filling and emptying of two contrast media (Level 2), BaSO_4_and (SMAT, in bearded dragons.

Part of digestive tract	pTP1	pTP2	p(TP2-TP1)
**Oropharynx**	1.000	0.857	0.857
**Esophagus**	0.765	1.000	1.000
**Stomach**	0.066	0.058	0.079
**Small intestine**	0.010	0.255	0.460
**Ampulla coli**	0.007	0.068	0.245
**Rectum**	0.534	0.316	0.861

pTP1 = p- values of time point 1; pTP2 = p- values of time point 2; p(TP2-TP1) = p- values of duration of retention period time as p(TP2-TP1). Level 2.

There was difference in the palatability and acceptance of the contrast agents, with the dragons readily accepting SMAT but not BaSO_4_. Regurgitation was also noted with BaSO_4_. The authors believe this difference was particular attributed to the taste of the contrast medium.

Table 9 provides an overview of the time courses for the contrast studies with barium sulfate (water—diluted Barilux suspension) and SMAT (Gastrografin), including the percentage and frequencies of animals at each time point (MTP). At 0.25 hours (15 minutes) after oral application of the contrast media, the small intestines contain SMAT in 66.7% (14/21) of the animals, while BaSO_4_ did not reach the intestines of any dragons by that time. After 0.5 hours (30 minutes), SMAT has entered the small intestines of 90.5% (19/21) of the bearded dragons, and BaSO_4_ is only observed in one (20%) of the animals. It required two full hours for SMAT to enter the small intestines of all the study animals and 8 hours for BaSO_4_ to reach the small intestines of all study animals. The ampulla coli was visible in all SMAT treated animals after 10 hours, while it required 36 hours for BaSO4 to reach the organ in 100% (5) of the animals. After 36 hours, 90.5% (19) of the lizards that received SMAT had contrast medium in the rectum, in comparison to 60% (3) of the animals that received BaSO4. Thus, in most of the animals, it was possible to visualize the entire digestive tract with SMAT within 36 hours (in 90.5% of the lizards), while 36 hours only allowed for the visualization of the entire gastrointestinal tract of 60% (3/5) of the bearded dragons with BaSO4 (Table 9).

**Table 9:** Time required for BaSO_4_ and SMAT contrast to appear in different sections of the bearded dragon gastrointestinal tract.

**MTP / POM (h)**	Contrast medium	Oropharynx	Esophagus	Stomach	Small intestine	Ampulla coli	Rectum
0	Barium sulfate	n = 5	n = 3	n = 2	n = 0	n = 0	n = 0
		TF = 100%	TF = 60%	TF = 40%	TF = 0%	TF = 0%	TF = 0%
0	(SMAT) Gastrografin	n = 21	n = 16	n = 10	n = 0	n = 0	n = 0
		TF = 100%	TF = 76.2%	TF = 47.6%	TF = 0%	TF = 0%	TF = 0%
0.25	Barium sulfate	n = 4	n = 5	n = 5	**n = 0**	n = 0	n = 0
		TF = 80%	TF = 100%	TF = 100%	**TF = 0%**	TF = 0%	TF = 0%
0.25	(SMAT) Gastrografin	n = 5	n = 8	n = 21	**n = 14**	n = 0	n = 0
		TF = 23.8%	TF = 38.1%	TF = 100%	**TF = 66.7%**	TF = 0%	TF = 0%
0.5	Barium sulfate	n = 3	n = 4	n = 5	**n = 1**	n = 0	n = 0
		TF = 60%	TF = 80%	TF = 100%	**TF = 20%**	TF = 0%	TF = 0%
0.5	(SMAT) Gastrografin	n = 3	n = 3	n = 21	**n = 19**	n = 0	n = 0
		TF = 14.3%	TF = 14.3%	TF = 100%	**TF = 90.5%**	TF = 0%	TF = 0%
0.75	Barium sulfate	n = 2	n = 3	n = 5	n = 1	n = 0	n = 0
		TF = 40%	TF = 60%	TF = 100%	TF = 20%	TF = 0%	TF = 0%
0.75	(SMAT) Gastrografin	n = 2	n = 2	n = 21	n = 19	n = 0	n = 0
		TF = 9.5%	TF = 9.5%	TF = 100%	TF = 90.5%	TF = 0%	TF = 0%
1	Barium sulfate	n = 1	n = 1	n = 5	n = 1	n = 0	n = 0
		TF = 20%	TF = 20%	TF = 100%	TF = 20%	TF = 0%	TF = 0%
1	(SMAT) Gastrografin	n = 2	n = 1	n = 21	n = 20	n = 0	n = 0
		TF = 9.5%	TF = 4.8%	TF = 100%	TF = 95.2%	TF = 0%	TF = 0%
2	Barium sulfate	n = 0	n = 0	n = 5	n = 3	n = 0	n = 0
		TF = 0%	TF = 0%	TF = 100%	TF = 60%	TF = 0%	TF = 0%
2	(SMAT) Gastrografin	n = 1	n = 1	n = 21	**n = 21**	n = 0	n = 0
		TF = 4.8%	TF = 4.8%	TF = 100%	**TF = 100%**	TF = 0%	TF = 0%
3	Barium sulfate	n = 0	n = 0	n = 5	n = 3	n = 0	n = 0
		TF = 0%	TF = 0%	TF = 100%	TF = 60%	TF = 0%	TF = 0%
3	(SMAT) Gastrografin	n = 0	n = 1	n = 21	n = 21	n = 1	n = 0
		TF = 4.8%	TF = 4.8%	TF = 100%	TF = 100%	TF = 4.8%	TF = 0%
4	Barium sulfate	n = 0	n = 0	n = 4	n = 3	n = 0	n = 0
		TF = 0%	TF = 0%	TF = 80%	TF = 60%	TF = 0%	TF = 0%
4	(SMAT) Gastrografin	n = 0	n = 0	n = 21	n = 21	n = 10	n = 2
		TF = 0%	TF = 0%	TF = 100%	TF = 100%	TF = 47.6%	TF = 9.5%
5	Barium sulfate	n = 0	n = 0	n = 4	n = 4	n = 0	n = 0
		TF = 0%	TF = 0%	TF = 80%	TF = 80%	TF = 0%	TF = 0%
5	(SMAT) Gastrografin	n = 0	n = 0	n = 20	n = 21	n = 13	n = 4
		TF = 0%	TF = 0%	TF = 95.2%	TF = 100%	TF = 61.9%	TF = 19.1%
6	Barium sulfate	n = 0	n = 0	n = 4	n = 4	n = 0	n = 0
		TF = 0%	TF = 0%	TF = 80%	TF = 80%	TF = 0%	TF = 0%
6	(SMAT) Gastrografin	n = 0	n = 0	n = 18	n = 21	n = 18	n = 6
		TF = 0%	TF = 0%	TF = 85.7%	TF = 100%	TF = 85.7%	TF = 28.6%
8	Barium sulfate	n = 0	n = 0	n = 4	**n = 5**	n = 1	n = 0
		TF = 0%	TF = 0%	TF = 80%	**TF = 100%**	TF = 20%	TF = 0%
8	(SMAT) Gastrografin	n = 0	n = 0	n = 13	n = 21	n = 20	n = 10
		TF = 0%	TF = 0%	TF = 61.9%	TF = 100%	TF = 95.2%	TF = 47.6%
10	Barium sulfate	n = 0	n = 0	n = 3	n = 5	n = 2	n = 0
		TF = 0%	TF = 0%	TF = 60%	TF = 100%	TF = 40%	TF = 0%
10	(SMAT) Gastrografin	n = 0	n = 0	n = 13	n = 20	**n = 21**	n = 13
		TF = 0%	TF = 0%	TF = 61.9%	TF = 95.2%	**TF = 100%**	TF = 61.9%
12	Barium sulfate	n = 0	n = 0	n = 3	n = 4	n = 3	n = 1
		TF = 0%	TF = 0%	TF = 60%	TF = 80%	TF = 60%	TF = 20%
12	(SMAT) Gastrografin	n = 0	n = 0	n = 12	n = 20	n = 21	n = 18
		TF = 0%	TF = 0%	TF = 57.1%	TF = 95.2%	TF = 100%	TF = 85.7%
24	Barium sulfate	n = 0	n = 0	n = 3	n = 3	n = 3	n = 2
		TF = 0%	TF = 0%	TF = 60%	TF = 60%	TF = 60%	TF = 40%
24	(SMAT) Gastrografin	n = 0	n = 0	n = 5	n = 11	n = 21	n = 20
		TF = 0%	TF = 0%	TF = 23.8%	TF = 52.4%	TF = 100%	TF = 95.2%
30	Barium sulfate	n = 0	n = 0	n = 2	n = 3	n = 4	n = 2
		TF = 0%	TF = 0%	TF = 40%	TF = 60%	TF = 80%	TF = 40%
30	(SMAT) Gastrografin	n = 0	n = 0	n = 1	n = 7	n = 21	n = 18
		TF = 0%	TF = 0%	TF = 4.8%	TF = 33.3%	TF = 100%	TF = 85.7%
36	Barium sulfate	n = 0	n = 0	n = 1	n = 3	**n = 5**	**n = 3**
		TF = 0%	TF = 0%	TF = 20%	TF = 60%	**TF = 100%**	**TF = 60%**
36	(SMAT) Gastrografin	n = 0	n = 0	n = 0	n = 1	n = 21	**n = 19**
		TF = 0%	TF = 0%	TF = 0%	TF = 4.8%	TF = 100%	**TF = 90.5%**

Contrast medium, number and percentage of animals and frequency at measuring time point; MTP (OR point of measurement; POM) (in h) in the different parts of the digestive tract.

n = Number of animals; TF = Percent of total frequency (in %), (Level 1–4, equivalent to contrast medium visible).

**Fig 31:**
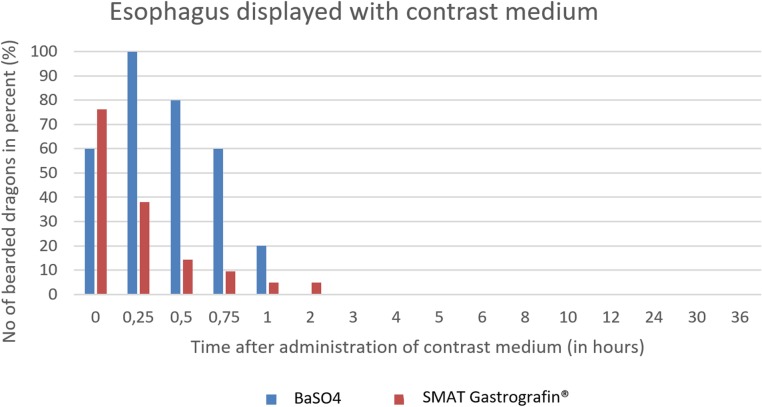
Radiographic visualization (OR imaging) with barium sulfate (BaSO_4_) (in blue) in comparison with SMAT (Gastrografin) (in red). Contrast passage time of the esophagus; percentage of animals and frequency at measuring time point; y = number of animals in percent (%) in which contrast media could be found in the esophagus; x = time point during the contrast passage (in hours).

The graphics (Figs 16–20) clearly show the differences of the passage times of both contrast media (SMAT and BaSO4). Fig 31 is displaying the time and number of animals after the administration of the two contrast media in the esophagus in comparison. The esophagus is displayed during the swallowing process. Fig 31 shows that the SMAT (Gastrografin) was rapidly swallowed by most of the animals and, in the case of a few, residues remained in the throat pouch, which were gradually transported into the stomach. The esophagus can be displayed with both contrast media best in between 0 minutes (immediately after the application of the contrast medium) and 1 hour.

**Fig 32:**
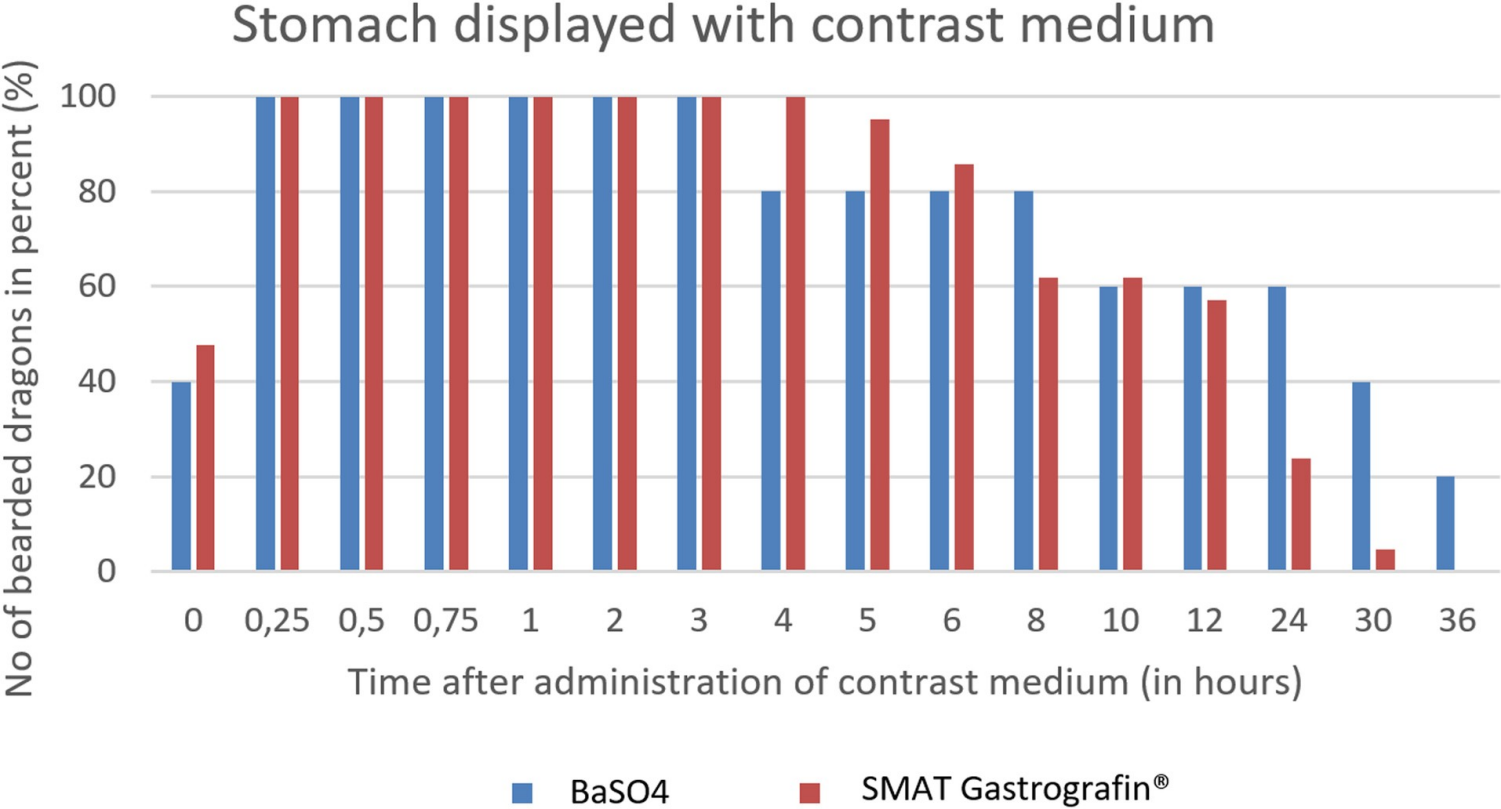
Radiographic visualization (OR imaging) with barium sulfate (BaSO_4_) (in blue) in comparison with SMAT (Gastrografin) (in red) contrast passage time of the stomach. Percentage of animals and frequency at measuring time point; y = number of animals in percent (%) in which contrast media could be found in the stomach; x = time point during the contrast passage (in hours).

The passage time of the stomach does not differ significantly between the two contrast agents (Fig 32). The stomach can be displayed with both contrast media best in between 15 minutes (0.25 hours) and 3 hours.

**Fig 33:**
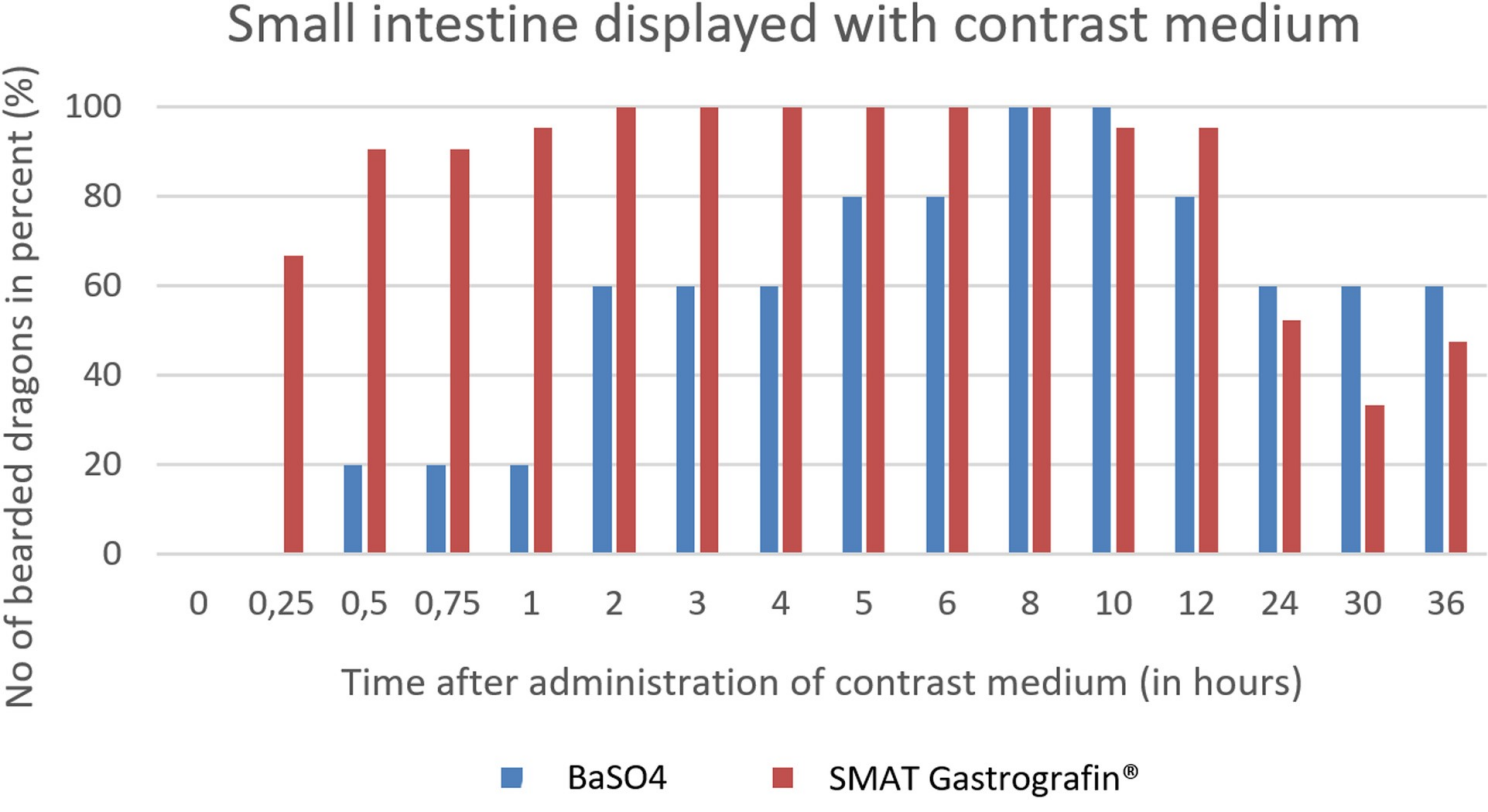
Radiographic visualization (OR imaging) with barium sulfate (BaSO_4_) (in blue) in comparison with SMAT (Gastrografin) (in red) contrast passage time of the small intestine. Percentage of animals and frequency at measuring time point; y = number of animals in percent (%) in which contrast media could be found in the small intestine; x = time point during the contrast passage (in hours).

In Fig 33, it can be seen that the small intestine can be seen in all of the bearded dragons as early as two hours post-SMAT (Gastrografin), but not until after 8 hours for BaSO_4_. Contrast medium can be seen in the small intestine for up to 36 hours after following the administration of both contrast media.

**Fig 34:**
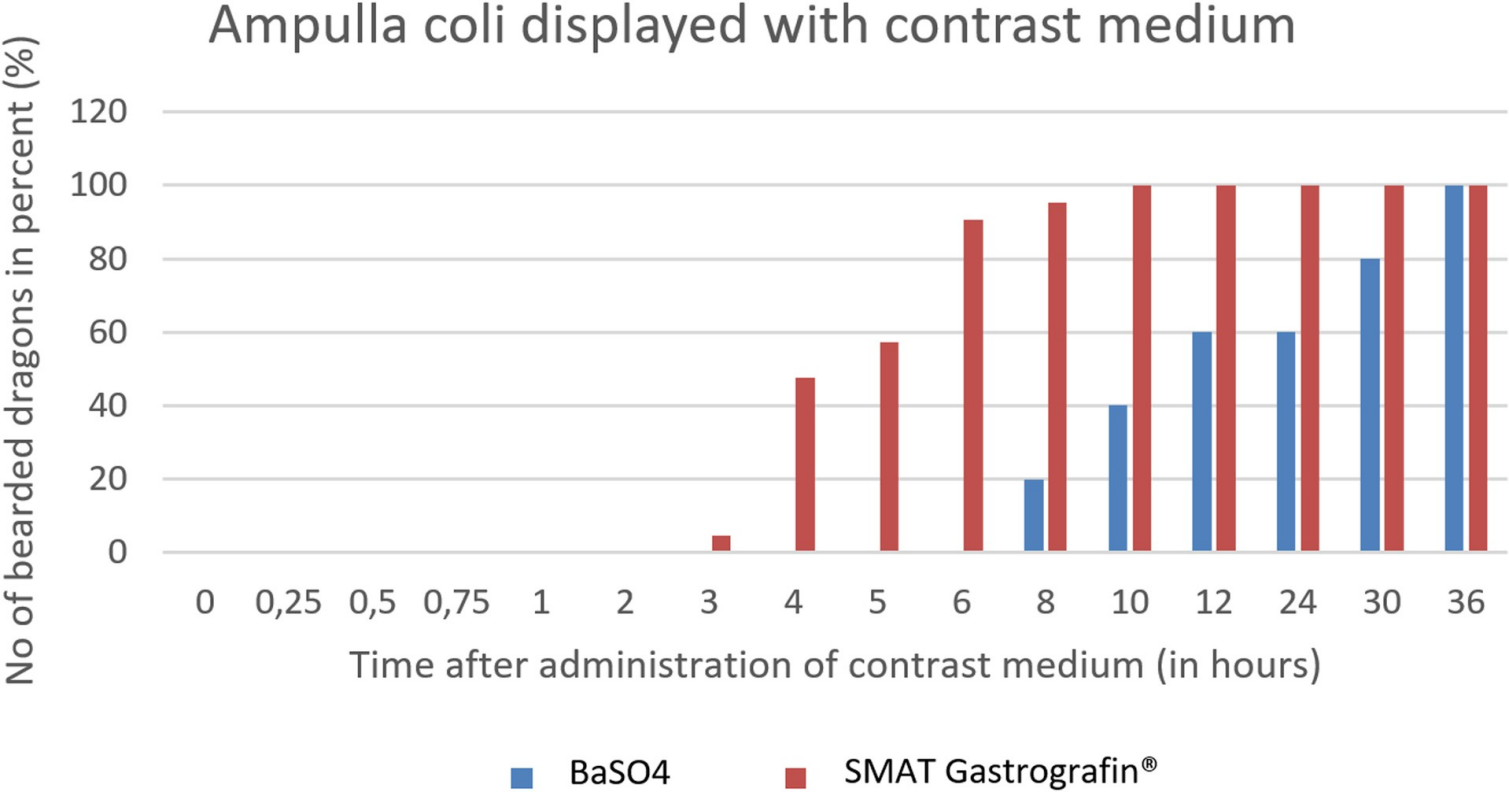
Radiographic visualization (OR imaging) with barium sulfate (BaSO_4_) (in blue) in comparison with SMAT (Gastrografin) (in red) contrast passage time of the ampulla coli. Percentage of animals and frequency at measuring time point; y = number of animals in percent (%) in which contrast media could be found in the ampulla coli; x = time point during the contrast passage (in hours).

The ampulla coli can be seen as early as 3 hours post-SMAT (Gastrografin), and achieved 100% contrast-positive status 10 hours after SMAT. BaSO_4_required 36 hours for treated animals to be 100% contrast positive in the ampulla coli (Fig 34). Contrast medium was observed in the ampulla coli for up to 36 hours post-dosing for both contrast media.

**Fig 35:**
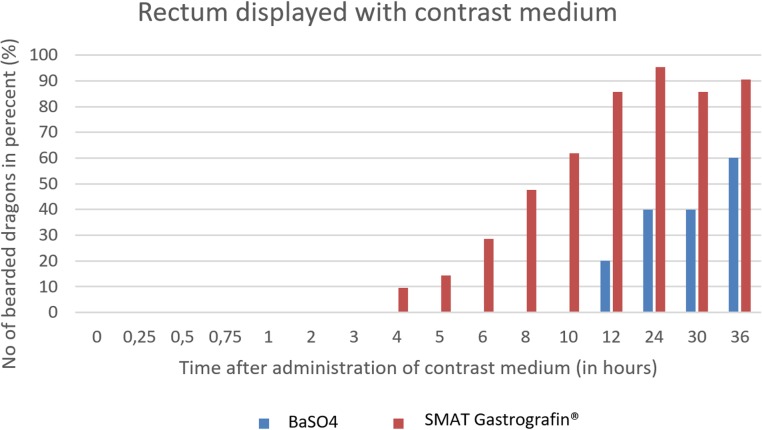
Radiographic visualization (OR imaging) with barium sulfate (BaSO_4_) (in blue) in comparison with SMAT (Gastrografin) (in red) contrast passage time of the rectum. Percentage of animals and frequency at measuring time point; y = number of animals in percent (%) in which contrast media could be found in the rectum; x = time point during the contrast passage (in hours).

SMAT (Gastrografin) could be seen in the rectum as early as 4 hours post-dosing and remained in the rectum for up to 36 hours (in 90,5% of the bearded dragons). BaSO4 could be seen in the rectum after 12 hours, and only 605 (3/5) of the animals remained contrast positive after 36 hours (Fig 35).

Comparison of the two contrast media for Level 1–4 is listed in Table 10 as p- values of time point 1 (pTP3), p-value of time point 2 (pTP4) and p-value of duration of retention period time as p(TP4-TP3).

**Table 10:** Comparison of the two contrast media filling times (Level 1–4) studies in bearded dragons with BaSO_4_ and SMAT.

Part of digestive tract	pTP3	pTP4	p(TP4-TP3)
**Oropharynx**	1.0000	0.0261	0.0065
**Esophagus**	0.1560	0.0136	0.0108
**Stomach**	0.8745	0.3370	0.3209
**Small intestine**	0.0009	0.3265	0.8686
**Ampulla coli**	0.0009	1.0000	0.1946
**Rectum**	0.0378	0.6488	0.0528

pTP3 = p- values of time point 1; pTP4 = p- values of time point 2; p(TP4-TP3) = p- values of duration of retention period time as p(TP4-TP3). Level 1–4.

**Fig 36:**
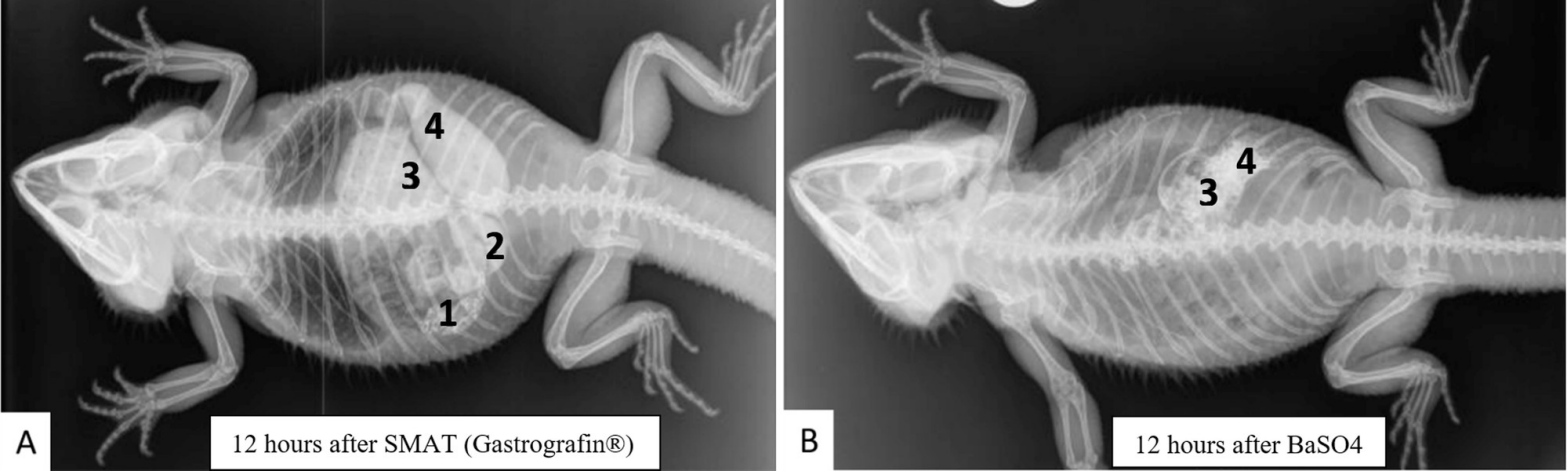
Same bearded dragon (Bearded dragon number 10), 12 hours after peroral application of contrast media. **A. Dorsoventral radiograph of bearded dragon number 10, 12 hours post-SMAT dosing.** Note the contrast in the stomach (1), small intestine (2), ampulla coli (3), and rectum (4). **B. Dorsoventral radiograph of bearded dragon number 10, 12 hours post- BaSO_4_ dosing**. Note that only the ampulla coli (3) and rectum (4) are clearly identified.

Overall, it can be concluded that the images with BaSO4 offer less information than the recordings with SMAT (Gastrografin), because BaSO4 was transported fractionated and compartmentalized. A good example for this is the bearded dragon number 10 in the 12-hour recording after application of contrast medium (Fig 36A and 36B). After administration of SMAT (Gastrografin), distal portions of the stomach, small intestine, ampulla coli, and cranial portions of the rectum can be well visualized, whereas the image 12 hours after BaSO4 application displays just a moderately well-filled ampulla coli with the transition of the contrast medium to the rectum. The uptake of barium sulfate would be difficult to interpret on its own, but with the administration of SMAT (Gastrografin), the display of almost the entire gastrointestinal tract is possible.

## Discussion

Gastroenterology represents an important specialty within clinical reptile medicine [37]. Knowledge of normal gastrointestinal anatomy is essential for interpreting reptile radiographs [5]; however, there is wide variability in the gastrointestinal anatomy of lizards and we lack evidence-based data to clarify these differences. The diversity in systems can depend on many factors, including the family, genus, and species, as well as their specific dietary needs. Therefore, mere comparisons of the anatomy of the alimentary canal between different groups of lizards may lead to false results. In this study we were able to identify unique gastrointestinal traits in bearded dragons, an omnivore, using gross and radiographic methods.

In contrast to other reptiles, such as chelonians, for which literature describing the anatomy of the digestive system exists,[38–48], the specific anatomy of this lizard species, including the alimentary canal, is rarely described in the literature, until today. Even if there are more recent studies describing the coelomic anatomy in conjunction with diagnostic imaging techniques for some other lizards, such as the green iguana (Iguana iguana) [49–51]; monitor species (*Varanus gouldii and V*. *indicus*) [50], and the leopard gecko (*Eublepharis macularius*) [32, 52], only a few studies are devoted to the coelomic anatomy including the digestive tract of the bearded dragon [2, 11, 25, 26].

The first part of the present work provides a detailed description of the gross anatomy and topography of the different sections of the gastrointestinal tract of bearded dragons, and thus serves as an additional source of evidence to the previously published reports on the anatomy of the gastrointestinal tract of various lizards [53–59], agamids, and bearded dragons [2, 25, 60]. In general, the reptilian gastrointestinal tract follows a similar pattern to that found in higher vertebrates, including an oropharynx, esophagus, stomach, small intestine, and large intestine [37]. In the present anatomical study, the different parts of the alimentary canal of the bearded dragons were referred to as the esophagus, ventriculus (stomach), duodenum, jejunum, ileum, ampulla coli, isthmus coli, rectum, and cloaca. Other authors refer to a stomach with fundus and pylorus, small intestine, cecum, colon, and cloaca, and mention a descending, a transversal, and an ascending stomach [2]. A recent study reported a duodenal bulb, which was equivalent to the “ascending stomach” mentioned by the former authors, and an ileo-cecal valve [25]. In our study, distinctions between the duodenum and the jejunum were made by the openings of the common bile and pancreatic ducts; the pancreas adjacent to the cranial part of the duodenum; the lumen size change between the sections; and the significantly longer mesentery of the jejunum. The ileum was differentiated by changes in color and lumen size. The subsequent section of the large intestine presents as an almost spherical dilatation, with a proximal and distal opening to the previous and following intestinal parts, respectively. Therefore, the section following the ileum is considered to be a part of the colon and referred to as “ampulla coli”. This is in contrast to other authors who termed this section as “transverse colon” [11] or, even more often and probably based on other species, as the “cecum” [2, 25, 26]. However, as the cecum is defined to be a “blindly ending section of the large intestine” [61], a “blindly ending cavity” [62], or “a sac or bodily cavity with only one opening” [63], the term “cecum” for this section of the large intestine would not be correct for bearded dragons. This opinion is undergirded by the presence of a small blind ending diverticulum of the ampulla coli in some bearded dragons. For this rudimentary section, the term “cecum” is correct. In the opinion of some authors, the small intestine is not clearly subdivided into separate sections and the large intestine (colon) consists of descending, transverse and ascending sections [11]. As the description of these structures in the former study was based on computed tomography images in transverse and sagittal projections, it is difficult to compare the so named structures with our own findings. The section following the isthmus coli is referred to as the rectum in this study because of its straight course; it was also shown in all dissected animals to terminate into the coprodeum. The straight course of this section of the large intestine could be seen radiographically with the contrast. Therefore, the term “rectum”, which is the Latin translation for straight, was chosen for this part of the digestive system in the bearded dragon and is in accordance with other authors [64]. Other investigators describe this section of the intestine not as rectum, but rather as colon [2, 25, 26] [2, 25, 26]; however, this does not appear to be appropriate based on the authors’ findings. In a recent contrast study, the gastrointestinal tract is divided into the esophagus, stomach, duodenum, jejunum, ileum, cecum, colon and cloaca [25].

The specific anatomical structures of the alimentary canal identified in the dissections provided a groundwork for the authors and allowed them to identify different sections of the alimentary canal on the radiographs in the contrast studies. A minimum of two radiographic images should be collected when evaluating a reptile patient [5]. Standard plain radiographs only provided limited assessment of the gastrointestinal tracts of the bearded dragons. Therefore, contrast was required to determine the specific radiographic location of the different sections of the alimentary canal [21].

Contrast agent studies are an important diagnostic tool for the detection of foreign bodies, circumferential proliferation, partial or complete intestinal obstruction or delayed gastric emptying. Gastrointestinal passage times vary with environmental temperature and diet in reptiles [21], as well as metabolic factors such as dehydration and hypocalcaemia [65]. Gastrointestinal passage times should be determined in healthy animals in order to identify physiological thresholds with pathological events. Therefore, in this study, the bearded dragons underwent a thorough clinical examination, an extensive blood analysis, as well as plain radiographs, to confirm they were healthy.

Gastrointestinal motility can be affected by a number of extrinsic and intrinsic factors [66]. The rate that food passes through the gastrointestinal tract can vary with not only the volume, type, and composition of food, but also with environmental and core body temperature [67]. Since intestinal motility and thus the passage time of a contrast agent is dependent on the environmental temperature in poikilothermic animals [21, 67], the ambient, body surface, and cloacal temperatures were measured over the course of this study. In the present study, the bearded dragons were housed in temperature zones between 17–34° C, with a daily fluctuation with lower temperatures at night than during the day. Some authors have reported ambient temperatures recorded monthly by weather stations in the natural ranges of *P*. *vitticeps*, and these temperatures ranged from sub-zero temperatures up to + 50° C, with mean values between 11–27° C [68]. Therefore, it can be assumed that the measured values in this study correspond to the temperature comfort zone of inland bearded dragons. Body surface and cloacal temperatures correlated well within both contrast study groups. Thus, one can postulate that the intestinal motility was not adversely affected by the measured temperatures in the present study comparing both contrast media studies.

The plain radiographic images were processed in two views in order to detect or rule out any pathology of the gastrointestinal tract [17]. Standard views in lizards are the dorsoventral and lateral images [69]. None of the plain radiographs in this study led to the exclusion of an animal due to obvious pathological changes. Several authors have suggested that plain survey radiographs of the digestive tract have little value [21, 25, 26, 70]. It has been suggested that even if gas, stones, or sand are ingested, a reliable statement about the topography of the intestinal tract cannot be made from either the laterolateral or dorsoventral views [65]. Therefore, contrast radiography is a necessary diagnostic tool for the evaluation of the gastrointestinal tract in daily clinical reptile practice [20]. Ultrasonography is a valuable tool for imaging the coelomic cavity of reptiles, especially in lizards and snakes [6], and has been extensively investigated in bearded dragons [2].

Several authors have suggested that BaSO4 can be used as a contrast medium in bearded dragons [25, 26], however, BaSO_4_is not without its potential side effects, including severe peritonitis if it leaks out of the gastrointestinal tract [71, 72]. Thickening of BaSO_4_ in the intestines can also lead to problems with a longer residence time, which may naturally occur because of slower motility rates in reptiles [34]. SMAT has proven to be very suitable as a simple oral contrast medium in small animals, unless it is used in debilitated animals [73]. The water-soluble substances can be absorbed from the bowel when in contact with the serosa and excreted by the kidneys without any expected damage. In cases of suspected constipation where leakage from the gastrointestinal tract can occur or in a foreign body case where surgery may be needed, BaSO_4_ should be avoided because of the risk of peritonitis. Because SMAT does not carry the same risk, it should be considered for these types of cases. The results of the current study suggest SMAT is safe, and provides better and faster results than BaSO_**4.**_ A previous study in red-eared slider turtles also found that the transit time of SMAT was faster than BaSO_4_ [34]. This is important because it shortens the length of time veterinary personnel need to image these animals. Additionally, in dehydrated animals, the BaSO_4_ can further slow transit times and the likelihood of a timely diagnosis.

Some of the differences noted in this study between the contrast agents may have been associated with the methods used. The BaSO_4_ dose of 9 ml / kg bodyweight was lower than previously reported by other authors [25–27, 70]. This dose was found to be insufficient to adequately image certain caudal sections of the small intestine, although we don’t believe it impacted passage time. The smaller volume of BaSO_4_ selected for the present work was based on the defensive behavior of the animals noted during oral administration of the contrast agent. Delivering a higher dose of BaSO_4_ was not possible without compulsory measures, which had to be rejected for reasons of animal welfare. In two other studies, BaSO_4_ was administered by esophageal gavage [25, 26]. This type of manipulation was not performed in our study because the use of a gastric or esophageal probe poses a potential risk for the animal, as it may cause injuries to structures of the upper digestive tract [74]. In a former study [25], the animals´ heads were kept upright during the administration of contrast medium and left in this position for another 30 seconds. Based on the authors’ experience with delivering the BaSO_4_in this study, we were also concerned about the risk of aspiration. Because of these concerns, we did not increase the BaSO_4_ dose. SMAT (Gastrografin) is certified as having less opacity and faster passage time than BaSO_4_ [34]. In the present work, these postulates were confirmed.

BaSO_4_was transported cumulatively and provided good density in the caudal sections of the gastrointestinal tract. SMAT was passed more steadily, which reduced the opacity but also resulted in illustrating the entire gastrointestinal tract with adequate density. In contrast to BaSO_4_, the continuous transport of SMAT (Gastrografin) also resulted in less radiographs. A former study on evaluating BaSO_4_ in bearded dragons cited the low cost, availability in practice, and good opacity in the digestive tract as reasons for choosing BaSO_4_ [25]. However, since bearded dragons are small-sized animals that need only small volumes of contrast media, the expense is negligible. In Germany, SMAT is readily available. Therefore, the only question is the comparable quality of the radiographic images between the two different contrast media. According to the authors, the slightly lower opacity of SMAT in the caudal section of the gastrointestinal tract is compensated by the excellent display of the entire gastrointestinal tract with only a few radiographic images.

In a preliminary study before the actual start of the present work, laterolateral images of several bearded dragons were taken by lateral rotation of the animals by 90° with the x-ray tube in a fixed position. For comparison, the animals were additionally x-rayed (OR radiographed) sitting in a physiological position with the x-ray tube rotated by 90° (OR degrees). According to other authors, the tube stand and design of the radiology area are important [75]. The aim of the preliminary (OR pilot) study before the actual start of the study was to find out whether the positioning of the animal or the beam path itself could be the problem causing superposition of the internal organs, a possible problem that had already been described by various other authors [25, 26]. In the preliminary study, the bearded dragons showed the intestinal sections at the physiological location, whereas the tilting of the bearded dragon by 90° for the laterolateral images forced a superposition of the visceral organs. Due to the tilting of the animals, the content of the coelom, which was anyway difficult to distinguish from each other on the laterolateral image, could now be assessed even less well overall. Subsequently, the laterolateral images of the animals in the current study were all taken in a physiological posture of the bearded dragons and all animals contributing to this study could thus be x-rayed without any further fixation. In almost all cases it was possible to position the extremities appropriately so that no superimpositions were visible on the pictures. The bearded dragons were usually cooperative during this positioning and so other aids, such as plastic containers, as used by other authors [26], could also be dispensed with. Pathological conditions of the esophagus in other animals such as birds are usually more easily detected on laterolateral radiographs [76]. The esophagus and stomach could be well visualized on the laterolateral images of all bearded dragons in this study. Further caudal intestinal sections could only be evaluated with the help of the dorsoventral image. The laterolateral radiation path alone would have led to misinterpretations. Other authors concluded the same [25, 26]. One author only took one lateral picture every 12 hours because the images were not expected to be very meaningful, but both authors recommended x-rays in both planes.

Digestibility coefficients in reptiles depend on the nutritional composition of the food being similar to that described in mammals. Although food intake is generally lower and digesta retention longer in reptiles than in mammals, digestive functions scale in a similar way in both clades, indicating universal principles [77]. In mammals, contrast medium studies are preferably performed on an empty stomach, as the intraluminal volume influences the passage time. In the present study, the bearded dragons were fed once daily in the morning, with a mixture of various vegetables and salads and the contrast agent study was completed after 36 hours. In one study, the animals were fasted and water was withdrawn 10 h prior to administration of the contrast medium [25]. In another contrast study with bearded dragons, the animals were provided food daily [26]. The results of both studies were similar. In the bearded dragons in the present study, SMAT was present in the rectum after 36 hours and the intestinal sections could be easily visualized. The results in the bearded dragons given BaSO_4_in the present study were less appealing, as the contrast did not pass through the entire gastrointestinal tract in any bearded dragon within 36 hours. In the current study, the small intestine was highlighted with BaSO_4_t, but only 40% (2/5) of animals emptied this section of their intestine within 36 hours. The ampulla coli and rectum also did not empty in the BaSO_4_ animals within 36 hours. Our results were different from another BaSO_4_study in bearded dragons, where complete passage of the contrast occurred within 36 hours in the 12 bearded dragons enrolled in the study [26], but similar to another where BaSO_4_ could be detected in all seven animals after 72 hours [25]. This variability could be due to differences between the studies or an anomaly associated with BaSO_4_. Regardless, these results suggest that BaSO_4_ may be inconsistent as a contrast agent in bearded dragons.

## Conclusions

This study demonstrated that it is possible to display the entire digestive tract (from oropharynx to cloaca) of inland bearded dragons under depicted standardized conditions with radiographs in dorsoventral and laterolateral views at pre-determined times. SMAT provided more consistent and timely images than BaSO_4_. SMAT is also safer to use in cases where the bowel may not be healthy. In the authors’ opinion, SMAT is preferable to BaSO_4_ as a contrast agent for of gastrointestinal studies in inland bearded dragons. Ensuring the bearded dragon has been fasted and is kept at an appropriate temperature will help ensure similar results to those reported here.
